# Identification of ancestry proportions in admixed groups across the Americas using clinical pharmacogenomic SNP panels

**DOI:** 10.1038/s41598-020-80389-9

**Published:** 2021-01-13

**Authors:** Guilherme Debortoli, Gilderlanio Santana de Araujo, Cesar Fortes-Lima, Esteban J. Parra, Guilherme Suarez-Kurtz

**Affiliations:** 1 Department of Anthropology, University of Toronto at Mississauga, Mississauga, ON Canada; 2 Program in Genetics and Molecular Biology, Federal University of Pará, Belém, Brazil; 3 Sub-Department of Human Evolution, Department of Organismal Biology, Evolutionary Biology Centre, Uppsala University, Uppsala, Sweden; 4 Instituto Nacional de Câncer and Rede Nacional de Farmacogenética, Rio de Janeiro, Brazil

**Keywords:** Genetic markers, Population genetics, Biomarkers

## Abstract

We evaluated the performance of three PGx panels to estimate biogeographical ancestry: the DMET panel, and the VIP and Preemptive PGx panels described in the literature. Our analysis indicate that the three panels capture quite well the individual variation in admixture proportions observed in recently admixed populations throughout the Americas, with the Preemptive PGx and DMET panels performing better than the VIP panel. We show that these panels provide reliable information about biogeographic ancestry and can be used to guide the implementation of PGx clinical decision-support (CDS) tools. We also report that using these panels it is possible to control for the effects of population stratification in association studies in recently admixed populations, as exemplified with a warfarin dosing GWA study in a sample from Brazil.

## Introduction

Many genetic variants associated with drug response show relatively large frequency differences between human populations^1–9^, and this has implications in terms of the clinical implementation of pharmacogenomics (PGx) to guide drug therapy. Several recent efforts have been made to evaluate the usefulness of PGx variants to infer biogeographical ancestry^3, 10, 11^. This is particularly important for studies in recently admixed populations in the Americas, which are characterized by varying admixture proportions from different continental groups^12–16^. Variation in admixture proportions between individuals creates population structure that can cause false positives in genetic association studies^17–20^. Bonifaz-Peña et al.^3^ developed a panel of 71 Ancestry Informative Markers (AIMs) extracted from the Affymetrix DMET Plus Platform to identify African, European and Native American contributions in populations across the Americas, and validated the panel using dense microarray data. Jackson et al.^11^ evaluated the capacity of the Affymetrix DMET Plus microarray to estimate population substructure and concluded, based on comparisons with genome-wide HapMap data, that it was an effective tool for ancestry inference in analyses including East Asian, African, European and Mexican samples. More recently, Hernandez et al.^10^ evaluated the ability of two clinical PGx panels, namely a Preemptive-PGx panel including 243 markers and a VIP panel including 122 SNPs, to estimate individual ancestry. The focus of Hernandez et al.^10^ paper was primarily to accurately identify ancestry in European and African American populations.

Obtaining accurate estimates of individual ancestry proportions using panels of PGx markers can have important applications for PGx-informed drug prescription. For genetic association studies in targeted genomic regions, inclusion of individual admixture proportions obtained with PGx panels in the statistical models can minimize the risk of false positive associations, which can be a problem in recently admixed populations. Additionally, PGx panels can be used to assign appropriate dosing algorithms for individual patients. As an example, Hernandez et al.^10^ have recently shown how estimates of individual ancestry obtained with PGx panels could be used to identify individuals with high African ancestry to whom a recently developed African–American-specific warfarin dosing algorithm could be applied^21^.

In this study, we evaluated the relative performance of three different PGx panels to infer individual ancestry in recently admixed populations in the Americas. We compared ancestry estimates obtained with dense genome-wide data with those obtained with the DMET AIMs panel developed by Bonifaz-Peña et al.^3^, as well as the Preemptive-PGx and VIP panels described by Hernandez et al.^10^. We also evaluated the extent to which estimates of individual ancestry obtained with the PGx panels correct for population structure in a Genome-Wide Association study (GWAs) of stable warfarin dose in Brazilian patients.

## Methods

### Genotype data

Dense genome-wide data and genotype data for the PGx panels (DMET, Preemptive-PGx and VIP) were extracted from 1000 Genomes Project (1KGP) and Human Genome Diversity Project (HGDP) samples^22, 23^ (Table 1).

**Table 1:** Reference and admixed populations cohorts obtained from 1000 Genomes Project and Human Genome Diversity Project.

Region	Group ID	Population	n
Africa	AFR	Esan from Nigeria	12
		Mende from Sierra Leona	12
		Yoruba from Ibadan Nigeria	12
		Gambians from Western Divisions in the Gambia	12
		Bantu samples from different regions of Subsaharan Africa	15
	Subtotal AFR		63
Europe	EUR	Utah Residents with Northern and Western European ancestry	15
		British from England and Scotland	15
		Iberians from Spain	15
		Toscani from Italy	15
	Subtotal EUR		60
East Asia	EAS	Southern Han Chinese	30
		Japanese from Tokyo, Japan	30
	Subtotal EAS		60
Native America	NAM	Pima from Mexico	13
		Maya from Mexico	13
		Piapoco and Curripaco from Colombia	6
		Karitiana from Brazil	12
		Surui from Brazil	8
	Subtotal NAM		52
Americas	AFR_ACB	African Caribbeans from Barbados	61
	AFR_ASW	Americans of African Ancestry in Southwest USA	96
	AMR_CLM	Colombians from Medellin Colombia	94
	AMR_MXL	Individuals of Mexican Ancestry from Los Angeles USA	64
	AMR_PEL	Peruvians from Lima Peru	85
	AMR_PUR	Puerto Ricans from Puerto Rico	104
	Total		739

The genome-wide panel included approximately 2.2 million markers obtained for the 1KGP and HGDP samples. Genomic reads of both datasets were previously mapped to the human GRCh38 reference assembly. All quality controls were performed with PLINK v1.9^24^. We extracted biallelic autosomal Single Nucleotide Polymorphisms (SNPs), removed SNPs with high genotyping error (> 1%), and excluded individuals with high missing genotyping rates (> 1%). After merging both datasets, we performed Hardy–Weinberg exact tests to exclude markers that failed a significance threshold of *p* < 1 × 10^–7^. For the LD-pruning process, we removed one SNP from a pair of SNPs if the LD was greater than the threshold of r^2^ = 0.2. For the LD-pruned HGDP-1KGP dataset, the final genome-wide panel included 2,180,911 SNPs.

For the three PGx panels, we extracted the targeted genotypes from the 1KGP and HGDP datasets. The DMET panel included 67 of the 71 ancestry-informative markers (Aims) previously reported by Bonifaz-Peña et al.^3^. The Preemptive-PGx and VIP panels comprised 219 of the 243 markers and 102 of 122 markers, respectively, reported by Hernandez et al.^10, 21^.

### Population structure analysis

The *smartpca* algorithm implemented in EIG v7.2.1 was used to perform the Principal Component Analysis (PCA) for the PGx panels and the genome-wide panel^25, 26^. The program ADMIXTURE was used to estimate individual ancestry proportions. Supervised and unsupervised analysis were performed with three and four-population models^27^.

The relative performance of the PGx panels to estimate admixture proportions was evaluated by the correlation of individual admixture proportions (R^2^ values) obtained with the genome-wide panel versus those obtained with each PGx panel. We also evaluated the differences in mean ancestry proportions obtained with the genome-wide panel and each PGx panel.

### Applicability of the PGx panels in a GWAs of warfarin dosing in Brazilian patients

In order to evaluate the ability of the PGx panels to correct for the effect of population stratification we used data collected in a previous GWAs of stable warfarin dosing in a cohort of Brazilian patients^28^. Briefly, this study included 180 individuals receiving low warfarin doses (≤ 20 mg/week) and 187 individuals receiving high warfarin doses (≥ 42.5 mg/week). The DNA samples were genotyped with Affymetrix Axiom Biobank array (Affymetrix, CA, USA). After quality control procedures, 314,000 markers were included in the statistical analyses. Association of genetic markers with warfarin dose (low vs. high-dose groups) was assessed using logistic regression under an additive model of inheritance. In order to evaluate the effect of population stratification in the association tests, we first carried out logistic regression analyses including as covariates sex, age, BMI, and amiodarone treatment, and estimated the genomic inflation factor (lambda). A second analysis was performed including admixture proportions estimated from genome-wide data and the PGx panels. Then, we evaluated population structure effects by observing genomic inflation factors for each logistic regression model. A lambda value of 1.0 indicates that there is no inflation in test statistics. Lambda values > 1.1 suggests strong influence of genetic structure or other design factors on the p-values^29^.

## Results

### Analysis of allele frequencies and Fst values

Supplementary Table 1 reports basic information about the markers included in the PGx panels, including rs#, chromosome, position, and allele frequencies in each parental group. Supplementary Table 2 provides mean Fst estimates for each pairwise population comparison. The DMET panel shows the largest mean Fst values between populations, except for the EAS-NAM comparison, for which the VIP panel has slightly higher mean Fst values than the DMET panel.

### PCA analyses

**Figure 1:**
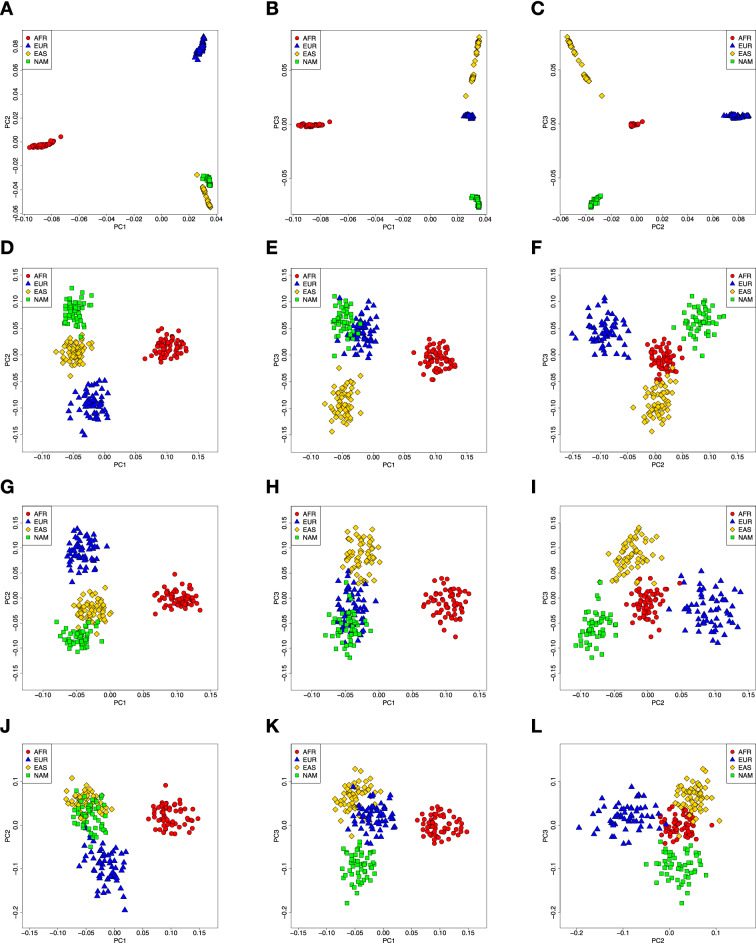
Principal Component Analysis of four major ancestry populations represented by AFR, EUR, EAS and AMR used as reference samples. (**A**–**C**) Genome-wide panel; (**D**–**F**) preemptive PGx panel; (**G**–**I**) DMET panel and (**J**–**L**) VIP panel.

As expected, the genome-wide panel provided very high resolution to differentiate the parental populations, due to the large number of markers included in the analysis (Fig. 1A–C). The PGx panels also provided reasonable separation of the parental groups. In this respect, the Preemptive-PGx panel (Fig. 1D–F) and to a lesser extent the DMET panel (Fig. 1G–I) provided more defined clusters than the VIP panel (Fig. 1J–L). PCA analyses including the parental groups and recently admixed population samples are provided in Supplementary Fig. 1. As expected, the admixed samples are primarily located between the clusters defined by the parental groups.

### Unsupervised and supervised ADMIXTURE analyses

The unsupervised ADMIXTURE analyses of the parental samples are presented in Supplementary Fig. 2. The genome-wide panel provides perfect discrimination between the individuals of each group (Supplementary Fig. 2A). All the individuals from each parental group belong to a different genetic cluster (AFR: orange, EUR: blue, EAS: yellow, and NAM, green). This is not the case for the three PGx panels (Supplementary Fig. 2B–D). In these analyses, individuals of each parental group have a predominant genetic cluster component, but also minor components from other clusters.

Next, we carried out supervised ADMIXTURE analysis including parental samples as reference groups and samples from the admixed populations of the Americas as test groups. These analyses provide estimates of the relative admixture proportions in individuals from the admixed samples. The results using four reference parental groups (AFR, EUR, EAS and NAM) are provided in Supplementary Fig. 3. The analyses using the genome-wide panel are in agreement with the trends observed in the PCA plots and highlight differences in the admixture proportions between the admixed samples (Supplementary Fig. 3A). Of note, the EAS genetic contribution is very low in all the admixed samples. The results obtained with the PGx panels are quite consistent with those observed with the genome-wide panel (Supplementary Fig. 3B–D), although it can be observed that the estimates of EAS genetic contributions obtained with the three PGx panels are higher than those obtained with the genome-wide panel.

**Figure 2:**
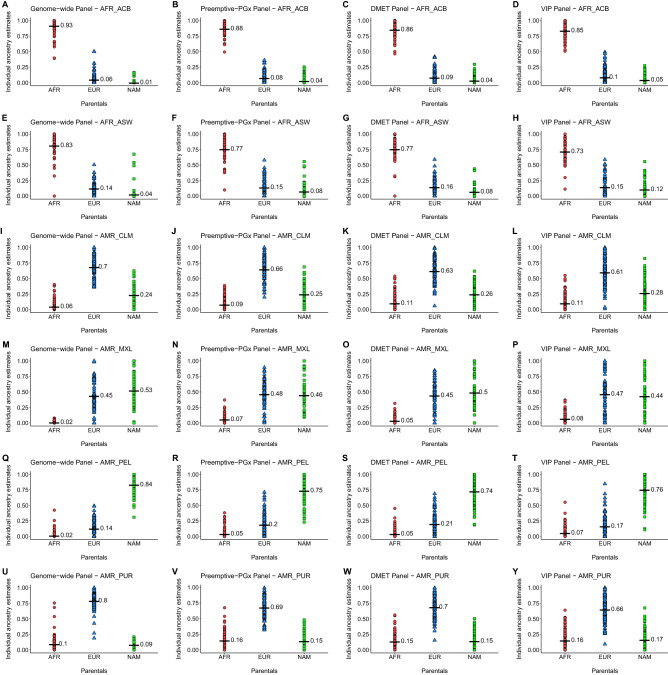
Individual estimates of the admixed populations for each ancestry with Genome-wide, Preemptive-PGx, DMET and VIP panels. (**A**–**D**) AFR_ACB; (**E**–**H**) AFR_ASW; (**I**–**L**) AMR_CLM; (**M**–**P**) AMR_MXL; (**Q**–**T**) AMR_PEL and (**U**–**Y**) AMR_PUR. Average denoted by black dash.

Given the very small EAS contributions observed in the admixed samples from the Americas (less than 1% in all samples), we repeated the supervised ADMIXTURE analyses using only three parental groups as reference samples (AFR, EUR, NAM). As shown in Supplementary Fig. 4, the results obtained with each PGx panel are very consistent with those observed with the genome-wide data. Figure 2 provide a different representation of these results, as plots of the distribution of individual ancestry proportions obtained by the genome-wide panel *versus* the proportions estimated using the PGx panels in each admixed sample.

### Correlation between genome-wide versus PGx panel admixture estimates

The relative performance of the PGx panels was measured by evaluating the correlation (R^2^ values) of individual admixture proportions obtained with the genome-wide panel and the PGx panels. The scatterplots of genome-wide vs. PGx panel estimates for each ancestry based on the six admixed populations analyzed in this study are depicted in Supplementary Fig. 5. The highest correlations with the genome-wide admixture estimates are observed for the Preemptive-PGx panel (R^2^ AFR = 0.95, R^2^ NAM = 0.89, R^2^ EUR = 0.86). The R^2^ values observed for the DMET panel are quite close to those of the Preemptive-PGx panel (R^2^ AFR = 0.95, R^2^ NAM = 0.85, R^2^ EUR = 0.83). The VIP panel shows lower R^2^ values than the other two panels, although they are still very high (R^2^ AFR = 0.89, R^2^ NAM = 0.75, R^2^ EUR = 0.73). The scatterplots corresponding to each admixed population are provided in Supplementary Figs. 6–11. The R^2^ values for individual populations are quite variable and depend heavily on the range of admixture observed for each ancestry. In general, the trends for each admixed population are very similar to those reported for the analyses including all admixed samples together, with the Preemptive-PGx panel typically showing the highest R^2^ values (although this is not always the case) and the VIP the lowest R^2^ values.

### PGx panels applicability to control for population stratification in a Brazilian sample

In order to evaluate the ability of the three PGx panels to correct for the effect of population stratification we used data collected in a previous GWAs of stable warfarin dosing in a sample from Brazil^28^ that included patients receiving low (≤ 20 mg/week) and high (≥ 42.5 mg/week) warfarin doses. The mean ancestry proportions in this sample are 78.5% EUR, 13.8% AFR and 7.7% NAM. Importantly, in this sample African ancestry is significantly associated with warfarin dosing (OR = 1.9, p = 0.007), so we would expect substantial genomic inflation in a GWAS without controlling for individual admixture proportions. This is exactly what we observed when carrying out the logistic regression analyses without individual admixture proportions (lambda = 1.18). When including individual admixture proportions obtained with the genome-wide panel in the model, the estimate of lambda is very close to 1 (lambda = 1.02). When including individual admixture proportions obtained with the three PGx panel, there is also a substantial reduction in inflation of test statistics (Preemptive-PGx panel, lambda = 1.02; DMET panel, lambda = 1.05; VIP panel, lambda = 1.06).

## Discussion

We carried out an exhaustive analysis of the performance of three PGx panels to estimate biogeographical ancestry: the DMET panel previously reported by Bonifaz-Pena et al.^3^, and the Preemptive-PGx and VIP panels recently described by Hernandez et al.^10^. It is important to note that one of the major goals of Hernandez et al.^10^ was to use these panels to identify individuals with ≥ 70% African ancestry, to whom an African–American-specific warfarin dosing algorithm could be applied. For validation of the Preemptive-PGx and VIP panels, Hernandez et al.^10^ used African, European and East Asian samples as reference groups, not including Native American samples to represent one of the major parental groups involved in the historical admixture process throughout the Americas. The present study included four parental groups, namely: African, European, Native American and East Asian, and we carried out ADMIXTURE analyses to evaluate the relative ancestry proportions in six admixed samples from the Americas. We observed that the East Asian contribution is very small in all these samples (lower than 1%), and focused our validation analyses mainly on models with three parental populations (African, European and Native American).

The PCA analyses show that the Preemptive-PGx panel and the DMET panel provide good discrimination of the four parental groups, which cluster with very little overlap in the plots. The VIP panel shows less discrimination than the other two panels (Fig. 1). Using supervised ADMIXTURE analyses based on three parental populations (AFR, EUR, NAM) we observed that the mean admixture proportions estimated with the PGx panels are very close to those obtained with the genome-wide panel (typically within 10% of the genome-wide estimates). The PGx panels typically underestimate the admixture proportions of the major parental group, and overestimate the admixture proportions of the minor parental groups (Fig. 2). The differences in mean admixture proportions tend to be higher with the VIP panel than with the other two PGx panels. The analysis of correlations of genome-wide and PGx panel individual admixture estimates provides more nuanced information (Supplementary Fig. 5). When considering all admixed samples in a combined analysis, the Preemptive-PGx and the DMET panels showed very good performances. For the Preemptive-PGx panel the R^2^ values were 0.95 (AFR), 0.89 (NAM) and 0.86 (EUR). The R^2^ values were almost as high for the DMET panel (R^2^ AFR = 0.95, R^2^ NAM = 0.85 and R^2^ EUR = 0.83), in spite of the fact that this panel has a smaller number of variants (67 markers) than the other two panels (219 markers for the Preemptive-PGx panel and 102 for the VIP panel). This is most probably driven by the approach used to select these markers, based on high allele frequency differences between the parental populations, which is reflected in higher mean FST values between parental populations for the DMET panel than for the Preemptive-PGx and VIP panels (Supplementary Table 2). The R^2^ values observed for the VIP panel, while smaller than for the other two panels, were still quite high (R^2^ AFR = 0.89, R^2^ NAM = 0.75 and R^2^ EUR = 0.73). Notably, the correlations in the estimates of African ancestry were extremely high for the three panels, confirming the results reported by Hernandez et al.^10^. Overall, our analysis indicates that the three PGx panels capture quite well the individual variation in admixture proportions observed in recently admixed populations throughout the Americas, and that the Preemptive-PGx and DMET panels tend to perform better than the VIP panel.

It is also relevant to discuss in more detail the results observed in the analysis of individual admixed populations (Supplementary Fig. 6), which clearly shows that the correlation of the genome-wide estimates with those obtained with the PGx panels is strongly dependent on the range of individual ancestry proportions present in the admixed population. Comparison of results for AFR_ASW and AFR_ACB is quite illustrative. The R^2^ values observed with the Preemptive-PGx panel for AFR and EUR ancestry for the AFR_ASW sample (AFR = 0.804 and EUR = 0.624) are substantially higher than those observed for the AFR_ACB sample (AFR = 0.322 and EUR = 0.370). This can be explained by the broader distribution of individual ancestry in the AFR_ASW than in the AFR_ACB sample (Fig. 2). Not surprisingly, the R^2^ values tend to be very low for the ancestral groups for which there are low average contributions with very limited ranges (Supplementary Figs. 6–11). In practice, this should have limited impact on the clinical utility of the PGx panels. As an example, in a hypothetical implementation of the approach described by Hernandez et al.^10^ for the selection of individuals with African ancestry ≥ 70% for application of an African–American-specific warfarin dosing algorithm, 86.9% of the AFR_ASW individuals and 91.7% of AFR_ACB individuals would have been selected by both the genome-wide and the Preemptive-PGx panel.

When performing association studies in recently admixed populations, an important concern is the possibility of obtaining inflated p-values due to the effects of population stratification^20, 30–32^. This is typically not an issue in GWAs studies based on microarray or whole genome data, as the individual ancestry estimates are very precise in this scenario and can be included in statistical models to control for the effects of stratification. However, when carrying out targeted association studies in limited genomic regions, it becomes more critical to ensure that there is an appropriate correction for population stratification. One possible strategy is to genotype panels including a limited number of AIMs, and use the estimates of individual ancestry obtained with these panels as covariates in the statistical models^14, 33, 34^. We compared the degree of inflation in the p-values of a GWAs study of warfarin dosing in a Brazilian sample^28^ using no individual admixture estimates in the statistical models, or alternatively including estimates of ancestry derived from a genome-wide panel or the PGx panels. This sample is perfectly suited for this analysis, as African ancestry shows a very strong association with high warfarin dosing (p = 0.007), in agreement with data indicating that, on average, individuals of African ancestry require higher warfarin doses than individuals of European ancestry^35–38^. As expected, if ancestry is not included in the logistic regression models, there is substantial genomic inflation (lambda = 1.18). In contrast, when including genome-wide estimates of individual ancestry in the logistic regression the estimates of lambda are reduced dramatically (genome-wide estimate, lambda = 1.02; Preemptive-PGx panel, lambda = 1.02; DMET, lambda = 1.05; VIP, lambda = 1.06). In summary, the three panels reduced significantly the inflation of test statistics.

In conclusion, our analysis of the DMET, Premptive-PGx and VIP panels highlight their usefulness for several PGx applications. We showed that these panels can provide reliable information about biogeographic ancestry. This information can be used to guide the implementation of PGx clinical decision-support (CDS) tools, as described by Hernandez et al.^10^. Overall, when considering how well the three PGx panels capture individual admixture proportions, the Preemptive-PGx and the DMET panels show the best performances, and the VIP panel provides less discrimination of the parental populations. Finally, we also show that using these panels it is possible to control for the effects of population stratification in association studies in recently admixed populations, as exemplified with a warfarin dosing GWAs study in Brazilian patients.

## Supplementary Information

Supplementary Figures.

Supplementary Tables.

## Data Availability

The datasets analysed in the current study are available from the corresponding authors upon reasonable request.
