# Supplementary material for: Identification of ancestry proportions in admixed groups across the Americas using clinical pharmacogenomic SNP panels
Source: Sci Rep. 2021 Jan 13;11:1007. doi: 10.1038/s41598-020-80389-9 (PMC7806998; doi:10.1038/s41598-020-80389-9)
Supplement: Supplementary file 1 — Supplementary Figures. [file 41598_2020_80389_MOESM1_ESM.pdf]

Identification of ancestry proportions in admixed groups across the Americas using clinical pharmacogenomic SNP panels.

Guilherme Debortoli[1*], Gilderlanio Santana de Araujo[2*], Cesar Fortes Lima[3], Esteban J. Parra[1#], Guilherme Suarez-Kurtz[4#].

1. *Department of Anthropology, University of Toronto at Mississauga, Mississauga, ON, Canada*
2. *Program in Genetics and Molecular Biology, Federal University of Pará, Belém, Brazil*
3. *Sub-department of Human Evolution, Department of Organismal Biology, Evolutionary Biology Centre, Uppsala University, Uppsala, Sweden*
4. *Instituto Nacional de Câncer and Rede Nacional de Farmacogenética, Rio de Janeiro, Brazil*

[*]These authors equally contributed to the manuscript

[#]Corresponding authors.

Guilherme Suarez-Kurtz, MD, PhD
Instituto Nacional de Câncer, Coordenação de Pesquisa - CPQ.
Rua André Cavalcanti 37, Rio de Janeiro, RJ – Brazil
Zip Code: 20231050 - Phone: +55 21 32076502
E-mail: kurtz@inca.gov.br

Esteban J. Parra, PhD

Department of Anthropology

University of Toronto at Mississauga

Mississauga, ON L5L 1C6

E-mail: esteban.parra@utoronto.ca

Supplementary Figure 1. Principal Component Analyzes including parental groups and recently admixed samples. **A** – genome-wide panel; **B** – Preemptive-PGx panel; **C** – DMET panel and **D** – VIP panel.

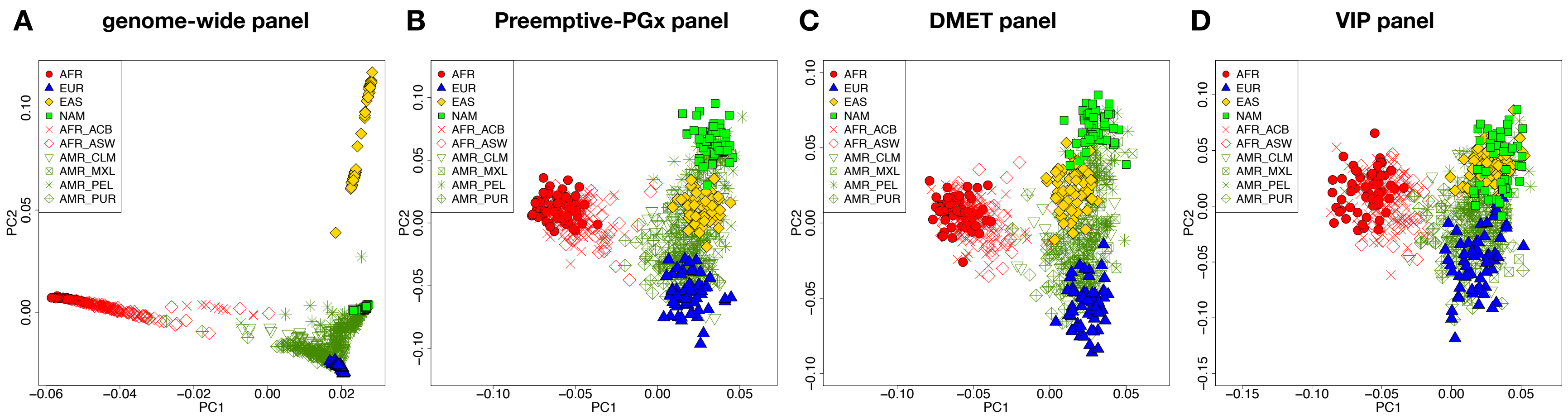

Supplementary Figure 2. Unsupervised ADMIXTURE Analysis for the four parental major global populations. **A** – genome-wide panel; **B** – Preemptive-PGx panel; **C** – DMET panel and **D** – VIP panel.

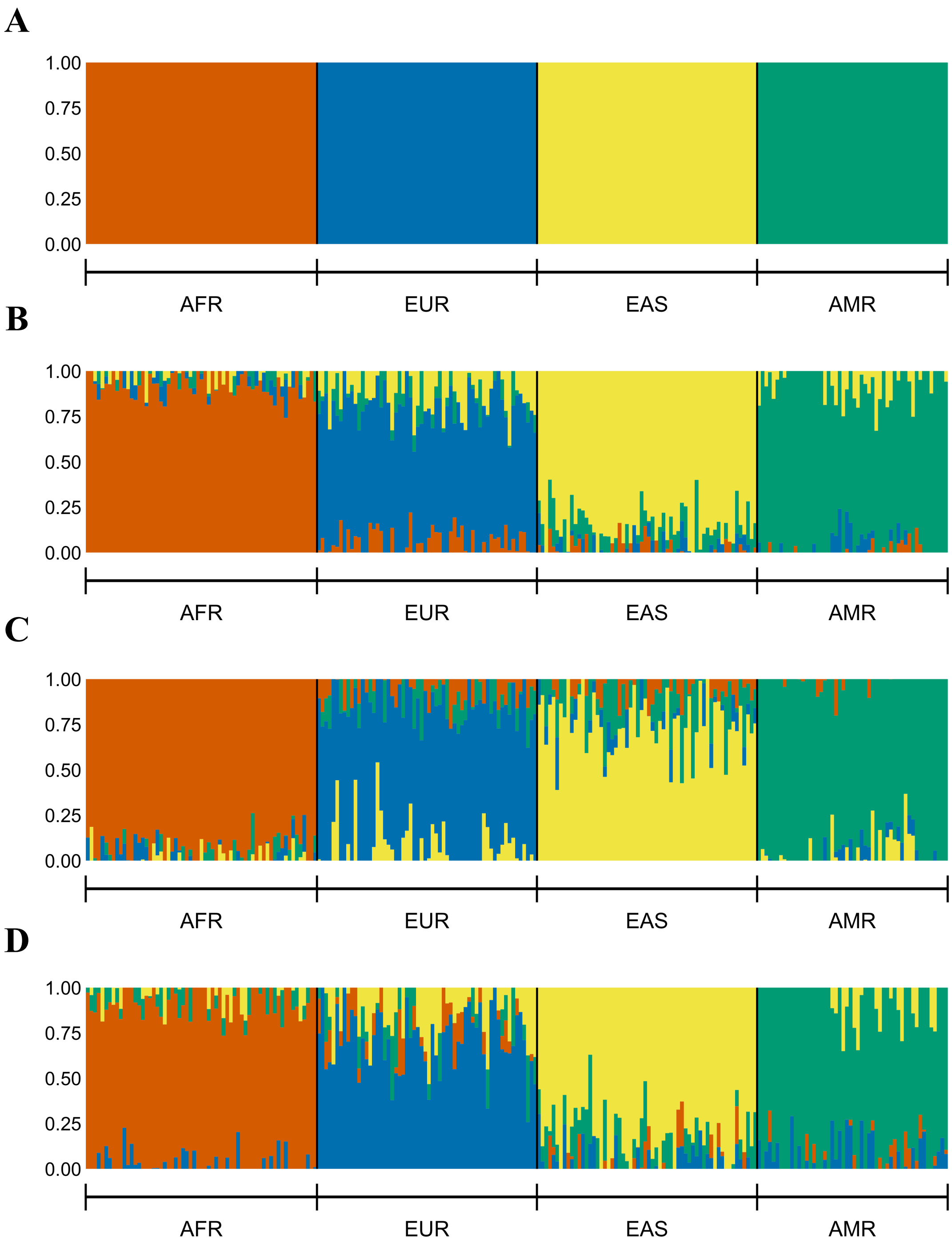

Supplementary Figure 3. Supervised ADMIXTURE Analysis of the admixed AMR, ASW and ACB samples (using 4 parentals). **A** – genome-wide panel; **B** – Preemptive-PGx panel; **C** – DMET panel and **D** – VIP panel.

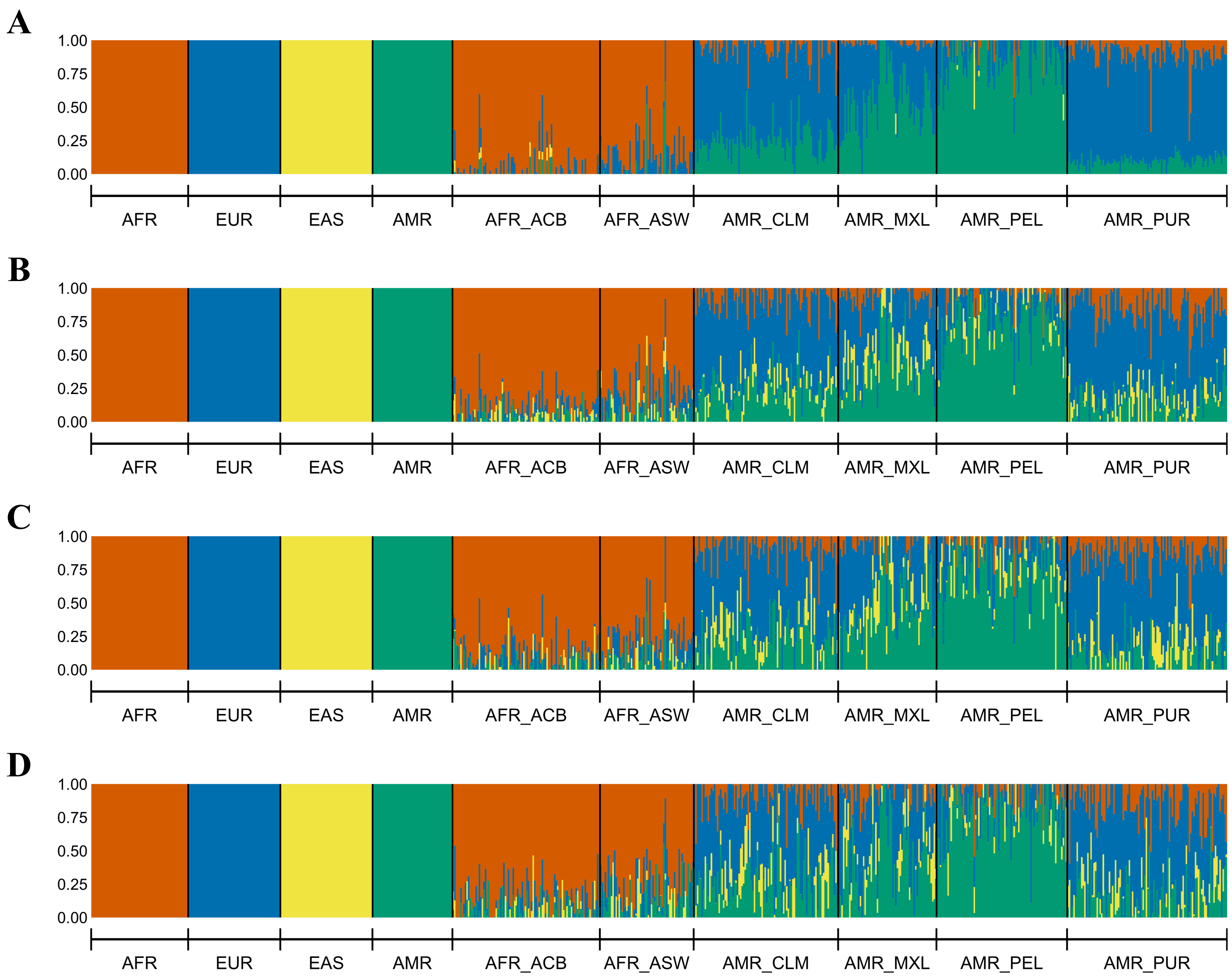

Supplementary Figure 4. Supervised ADMIXTURE Analysis of the admixed AMR, ASW and ACB samples (using 3 parentals). **A** – genome-wide panel; **B** – Preemptive-PGx panel; **C** – DMET panel and **D** – VIP panel.

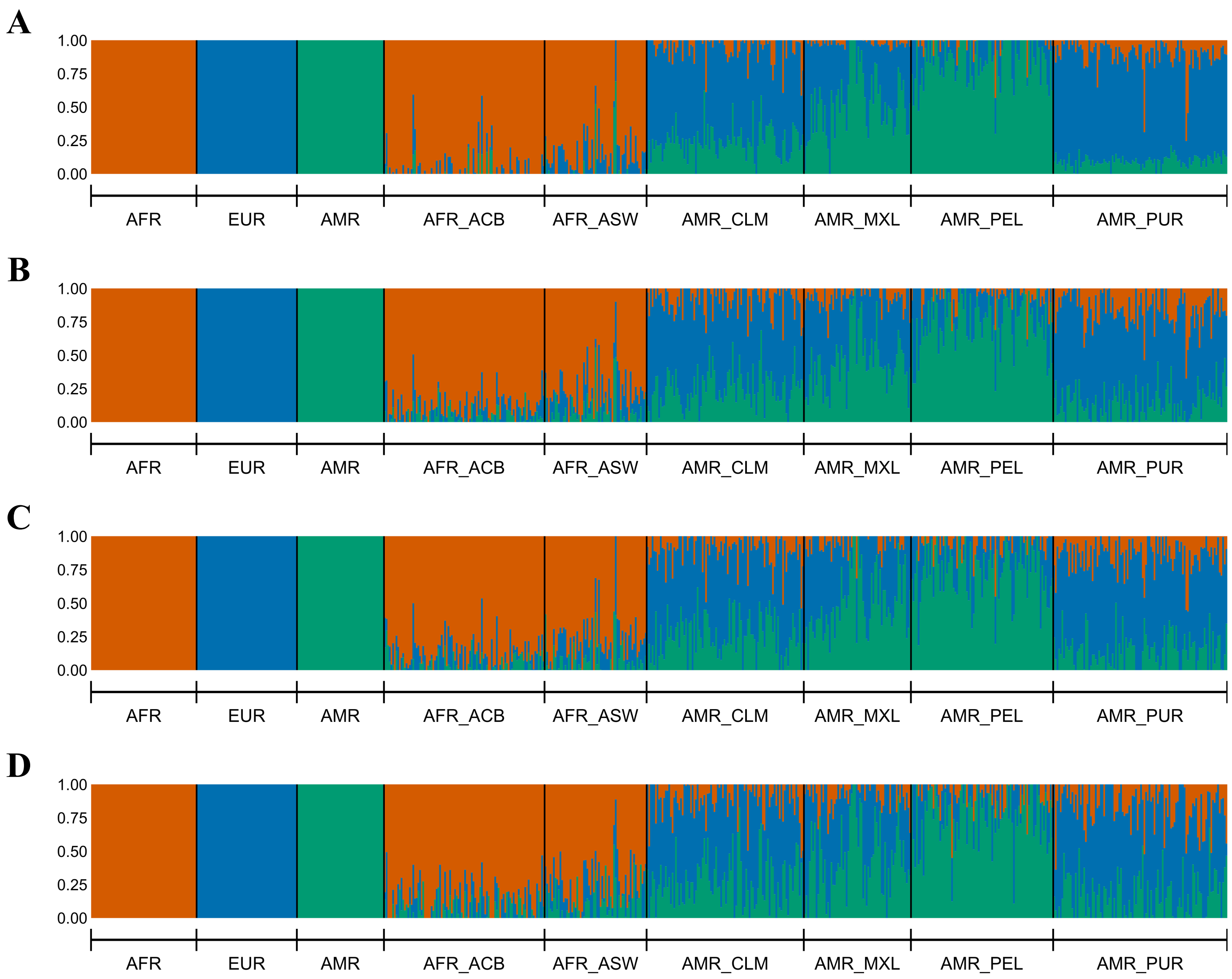

Supplementary Figure 5. Genome-wide vs. PGx panels estimates for each ancestry based on the six admixed populations analyzed. **A-C** – AFR ancestry; **D-E** – EUR ancestry and **F-H** – NAM ancestry.

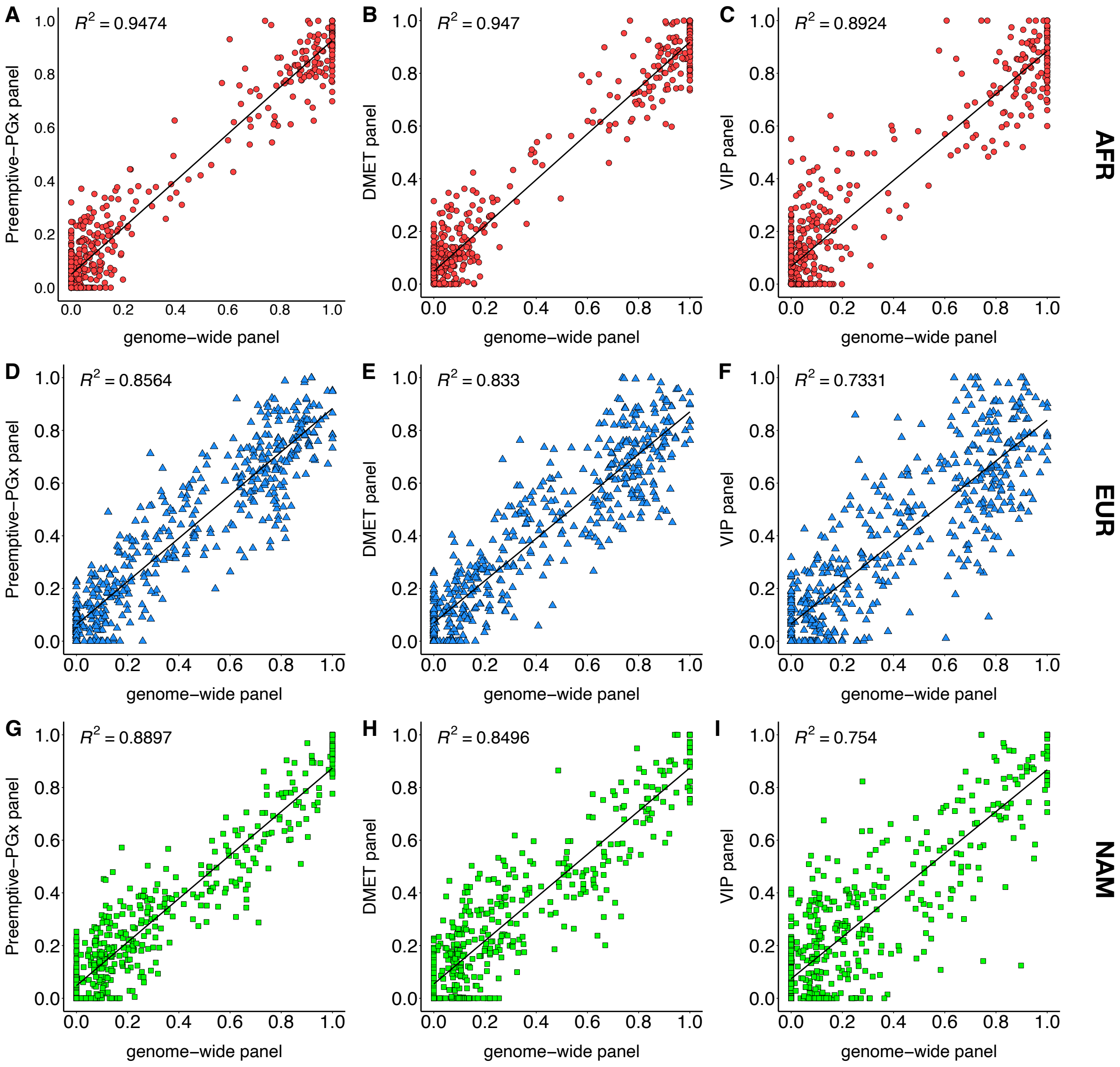

Supplementary Figure 6. Genome-wide vs. PGx panels estimates for each ancestry based on the AFR_ACB admixed population. **A-C** – AFR ancestry; **D-E** – EUR ancestry and **F-H** – NAM ancestry.

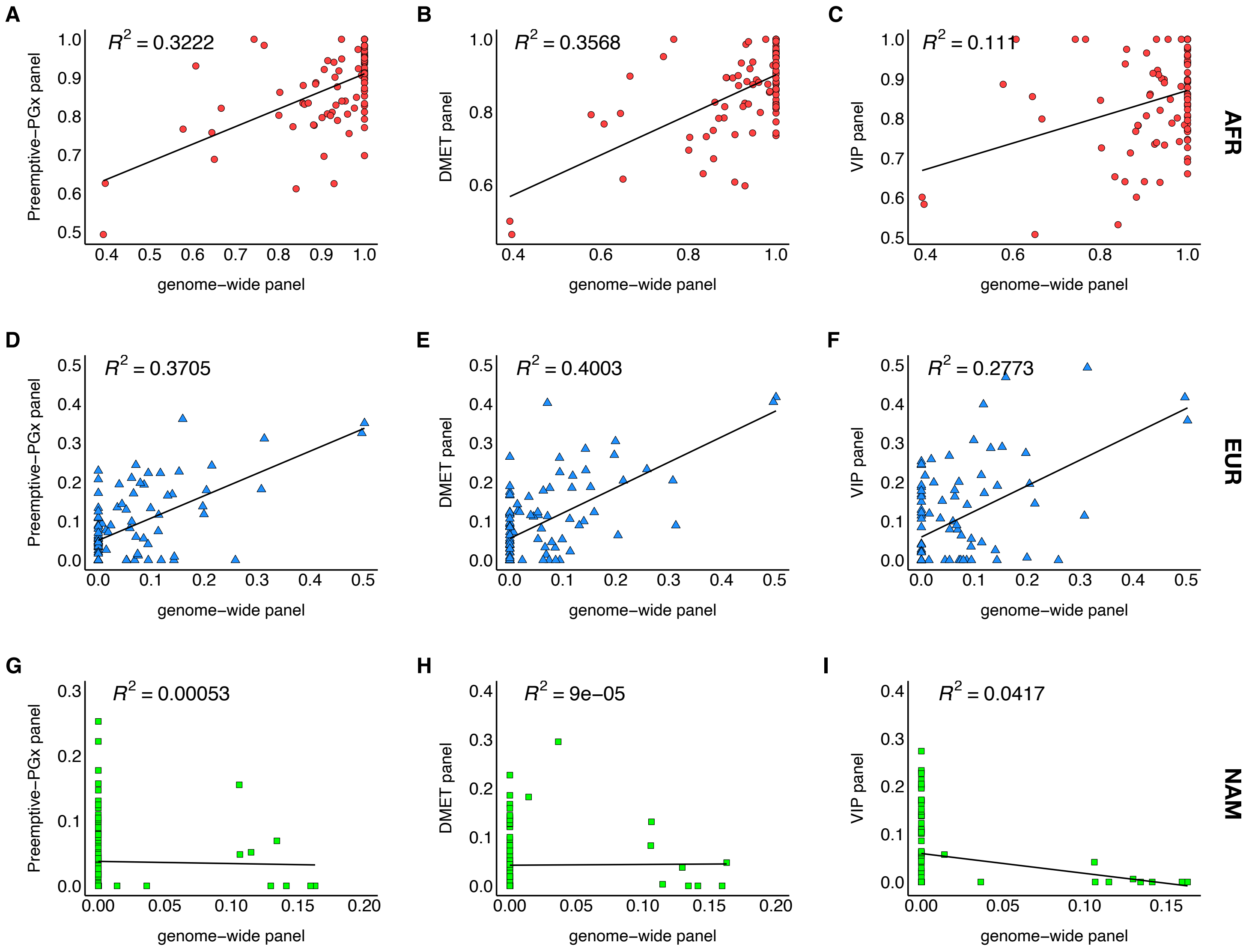

Supplementary Figure 7. Genome-wide vs. PGx panels estimates for each ancestry based on the AFR_ASW admixed population. **A-C** – AFR ancestry; **D-E** – EUR ancestry and **F-H** – NAM ancestry.

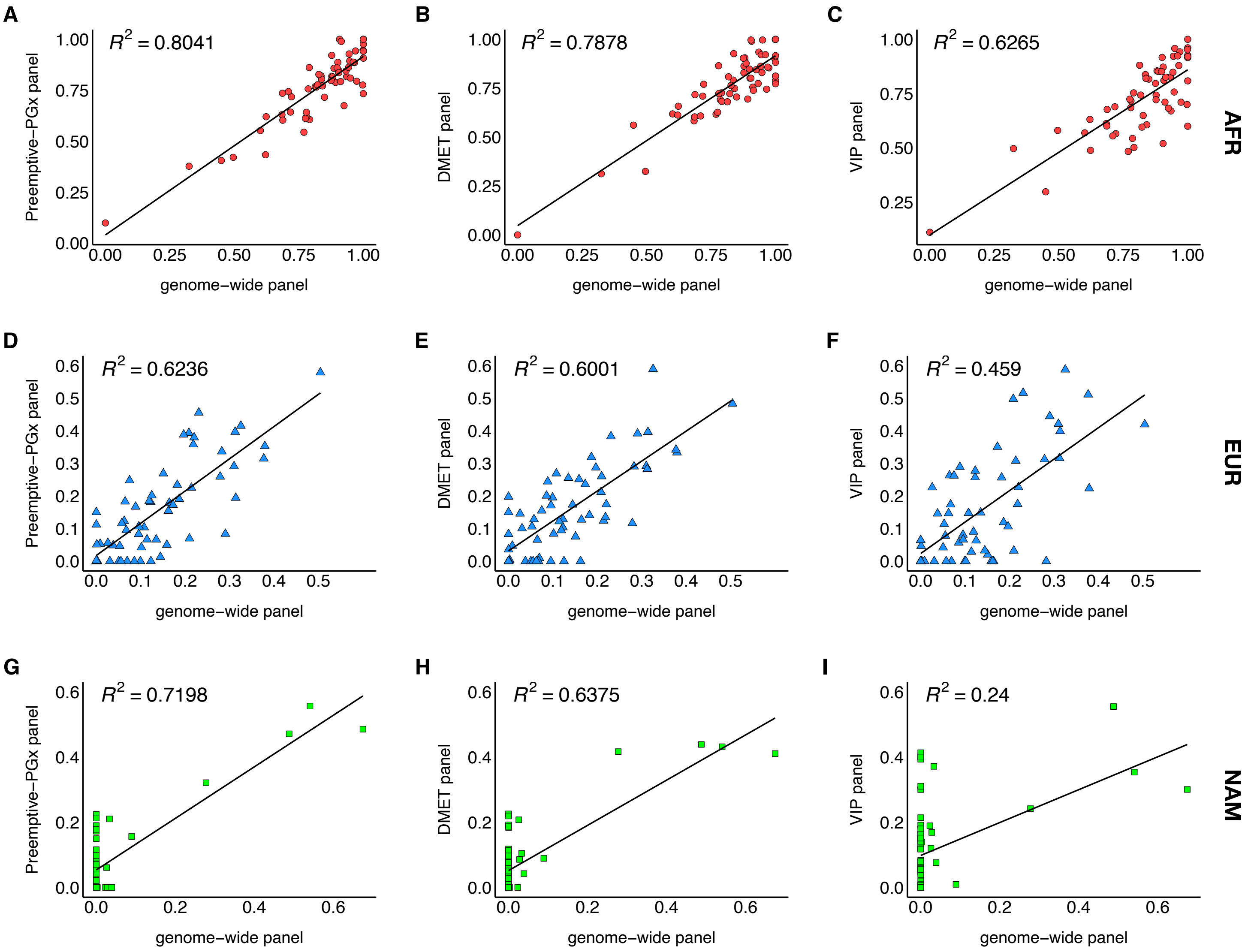

Supplementary Figure 8. Genome-wide vs. PGx panels estimates for each ancestry based on the AMR_CLM admixed population. **A-C** – AFR ancestry; **D-E** – EUR ancestry and **F-H** – NAM ancestry.

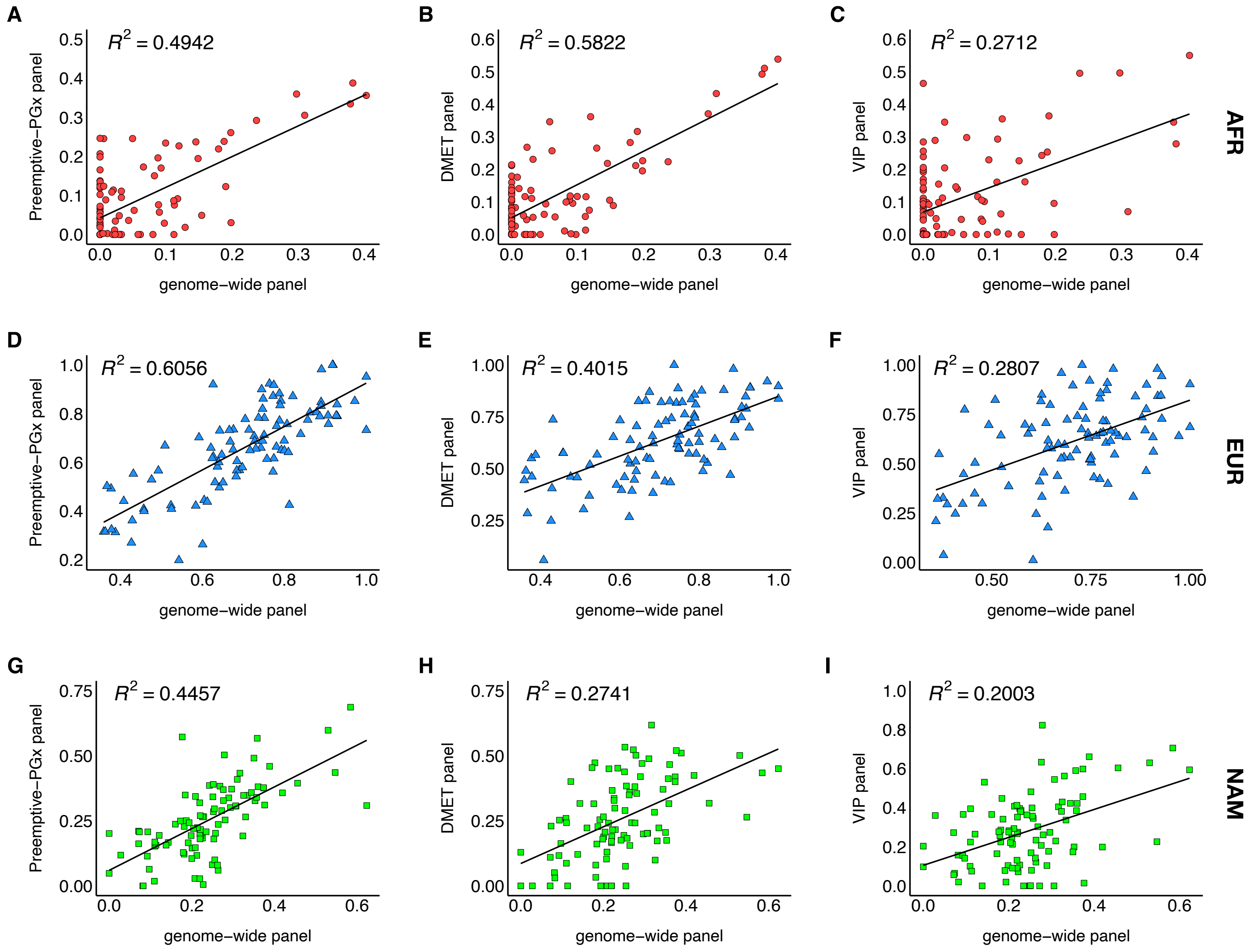

Supplementary Figure 9. Genome-wide vs. PGx panels estimates for each ancestry based on the AMR_MXL admixed population. **A-C** – AFR ancestry; **D-E** – EUR ancestry and **F-H** – NAM ancestry.

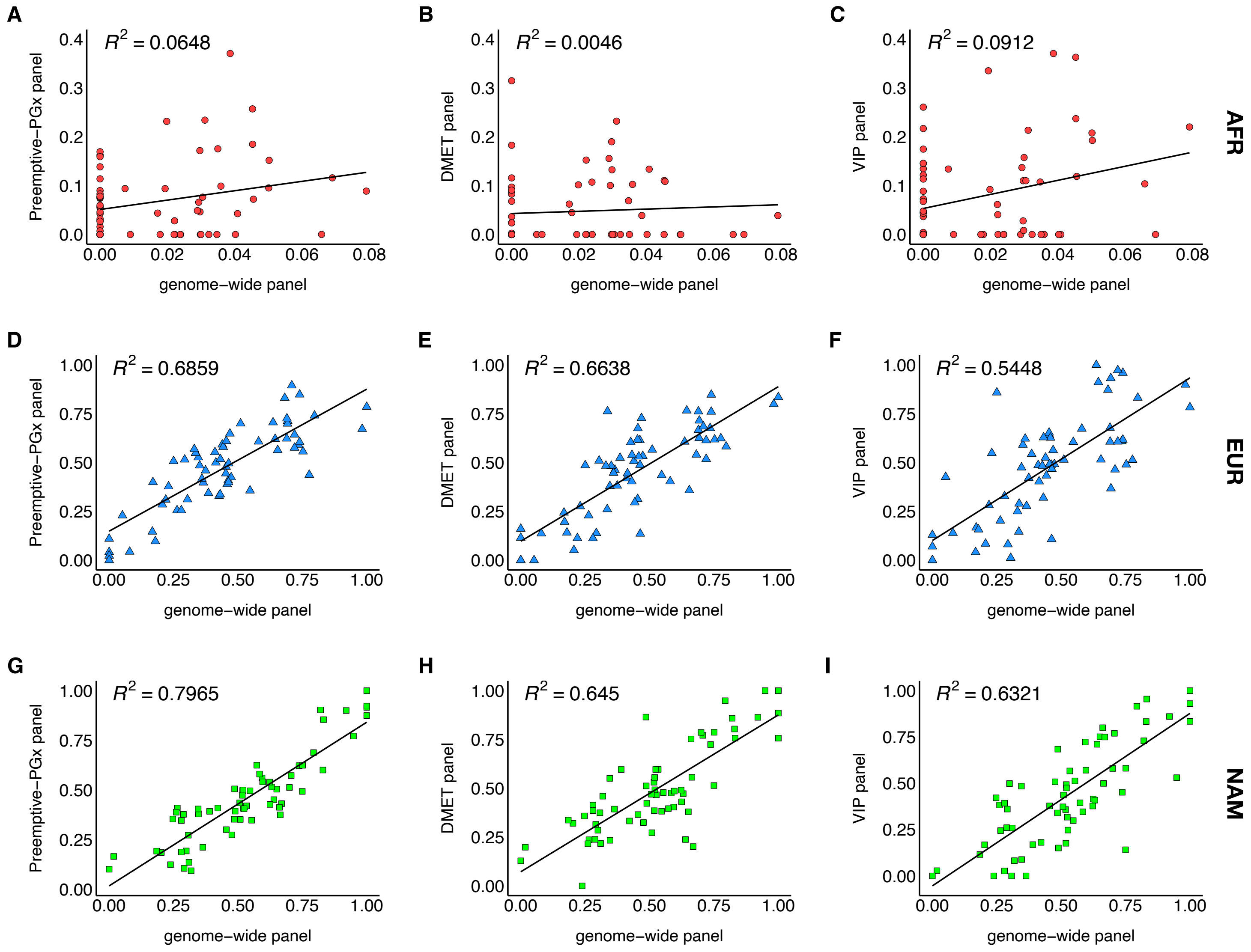

Supplementary Figure 10. Genome-wide vs. PGx panels estimates for each ancestry based on the AMR_PEL admixed population. **A-C** – AFR ancestry; **D-E** – EUR ancestry and **F-H** – NAM ancestry.

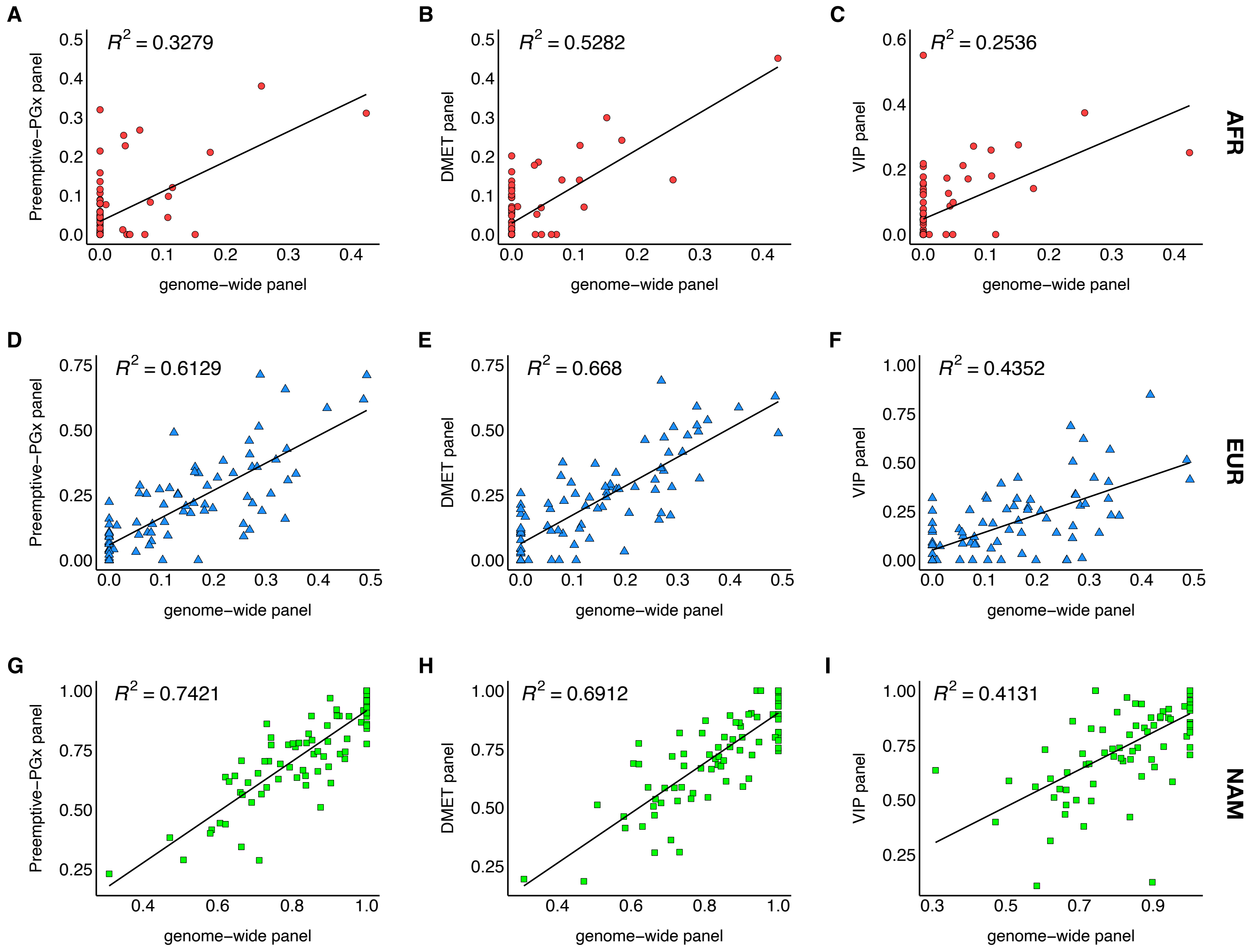

Supplementary Figure 11. Genome-wide vs. PGx panels estimates for each ancestry based on the AMR_PUR admixed population. **A-C** – AFR ancestry; **D-E** – EUR ancestry and **F-H** – NAM ancestry.

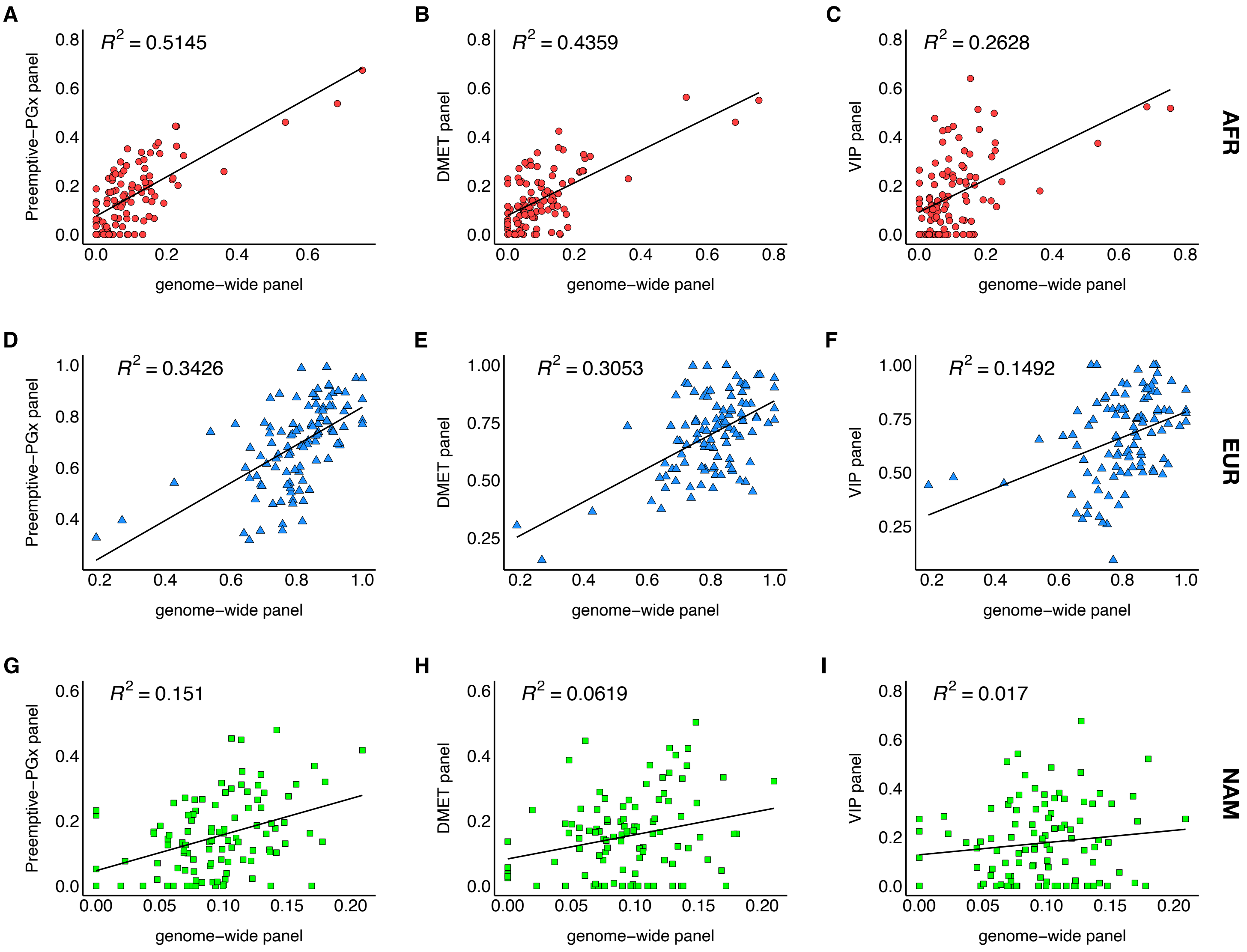

Supplementary information is available at The Pharmacogenomics Journal's website.
