# Supplementary material for: Identification of ancestry proportions in admixed groups across the Americas using clinical pharmacogenomic SNP panels
Source: Sci Rep. 2021 Jan 13;11:1007. doi: 10.1038/s41598-020-80389-9 (PMC7806998; doi:10.1038/s41598-020-80389-9)
Supplement: Supplementary file 2 — Supplementary Tables. [file 41598_2020_80389_MOESM2_ESM.docx]

| **Supplementary Table 1. Information on the biomarkers of the PGx panels** | | | | | | | |
| --- | --- | --- | --- | --- | --- | --- | --- |
| **Preemptive-PGx panel** | | | | | | | |
| CHR | SNP | A1 | A2 | AFR | EUR | EAS | AMR |
| 1 | rs1801131 | G | T | 0.1190 | 0.3083 | 0.2000 | 0.0673 |
| 1 | rs5065 | G | A | 0.4286 | 0.1500 | 0.0167 | 0.0000 |
| 1 | rs5063 | T | C | 0.0635 | 0.0333 | 0.1000 | 0.0096 |
| 1 | rs1061622 | G | T | 0.1270 | 0.2083 | 0.1500 | 0.0385 |
| 1 | rs4646091 | C | T | 0.4921 | 0.2000 | 0.0833 | 0.0673 |
| 1 | rs2072671 | C | A | 0.0556 | 0.3750 | 0.1333 | 0.4519 |
| 1 | rs1048977 | T | C | 0.3810 | 0.3167 | 0.2500 | 0.4135 |
| 1 | rs1137101 | A | G | 0.3810 | 0.5000 | 0.1500 | 0.4038 |
| 1 | rs67376798 | A | T | 0.0000 | 0.0250 | 0.0000 | 0.0000 |
| 1 | rs1801159 | C | T | 0.1746 | 0.1833 | 0.1500 | 0.3846 |
| 1 | rs2297595 | C | T | 0.0318 | 0.1000 | 0.0083 | 0.0000 |
| 1 | rs1801265 | G | A | 0.4841 | 0.2333 | 0.0583 | 0.1731 |
| 1 | rs17602729 | A | G | 0.0000 | 0.1250 | 0.0000 | 0.0000 |
| 1 | rs2297480 | T | G | 0.7143 | 0.7000 | 0.2417 | 0.2692 |
| 1 | rs2307418 | G | T | 0.0079 | 0.1083 | 0.0833 | 0.0096 |
| 1 | rs2307424 | A | G | 0.0952 | 0.3167 | 0.5917 | 0.5096 |
| 1 | rs1801274 | G | A | 0.5079 | 0.4917 | 0.2833 | 0.7404 |
| 1 | rs6025 | T | C | 0.0000 | 0.0167 | 0.0000 | 0.0000 |
| 1 | rs875326 | T | C | 0.3810 | 0.2000 | 0.0250 | 0.0865 |
| 1 | rs11240688 | A | G | 0.1508 | 0.1833 | 0.4083 | 0.3846 |
| 1 | rs11240594 | A | G | 0.1984 | 0.1917 | 0.5750 | 0.4231 |
| 1 | rs1051740 | C | T | 0.1746 | 0.2833 | 0.4833 | 0.4712 |
| 1 | rs2234922 | G | A | 0.3730 | 0.1500 | 0.0917 | 0.0000 |
| 1 | rs7079 | T | G | 0.0476 | 0.3000 | 0.1000 | 0.0000 |
| 1 | rs5051 | C | T | 0.0476 | 0.6333 | 0.1333 | 0.0673 |
| 1 | rs1805087 | G | A | 0.3333 | 0.1750 | 0.1833 | 0.2404 |
| 2 | rs676210 | A | G | 0.0714 | 0.2083 | 0.6417 | 0.2596 |
| 2 | rs1367117 | A | G | 0.0794 | 0.3500 | 0.1667 | 0.2500 |
| 2 | rs1056836 | C | G | 0.9048 | 0.4750 | 0.1167 | 0.0769 |
| 2 | rs11887534 | C | G | 0.1032 | 0.0667 | 0.0167 | 0.0769 |
| 2 | rs11676382 | G | C | 0.0000 | 0.0833 | 0.0000 | 0.0000 |
| 2 | rs1143634 | A | G | 0.1111 | 0.2417 | 0.0417 | 0.0000 |
| 2 | rs16944 | G | A | 0.4683 | 0.6500 | 0.6000 | 0.0962 |
| 2 | rs3812718 | T | C | 0.3413 | 0.4833 | 0.4750 | 0.4712 |
| 2 | rs5742909 | T | C | 0.0000 | 0.1083 | 0.1333 | 0.0192 |
| 2 | rs4673993 | C | T | 0.0635 | 0.3167 | 0.2917 | 0.4038 |
| 2 | rs1801278 | T | C | 0.0476 | 0.1000 | 0.0167 | 0.0000 |
| 2 | rs1042597 | G | C | 0.0318 | 0.2333 | 0.4500 | 0.3077 |
| 2 | rs17863762 | A | G | 0.0000 | 0.0167 | 0.0000 | 0.0000 |
| 2 | rs17868320 | T | C | 0.0238 | 0.0417 | 0.0000 | 0.0000 |
| 2 | rs72551330 | C | T | 0.0000 | 0.0083 | 0.0000 | 0.0000 |
| 2 | rs11692021 | C | T | 0.2619 | 0.3750 | 0.1833 | 0.0385 |
| 2 | rs4148323 | A | G | 0.0000 | 0.0000 | 0.1250 | 0.0385 |
| 3 | rs1801282 | G | C | 0.0000 | 0.1083 | 0.0500 | 0.0962 |
| 3 | rs2228001 | G | T | 0.2778 | 0.3917 | 0.3417 | 0.2308 |
| 3 | rs1800734 | A | G | 0.1905 | 0.3000 | 0.5667 | 0.3077 |
| 3 | rs1799977 | G | A | 0.0079 | 0.2667 | 0.0667 | 0.0096 |
| 3 | rs11706052 | G | A | 0.0079 | 0.1083 | 0.0500 | 0.0098 |
| 3 | rs1029871 | C | G | 0.1032 | 0.3583 | 0.4750 | 0.5481 |
| 3 | rs6280 | T | C | 0.1349 | 0.7000 | 0.7417 | 0.4038 |
| 3 | rs1801019 | C | G | 0.0873 | 0.1083 | 0.2250 | 0.4808 |
| 3 | rs5186 | C | A | 0.0238 | 0.3667 | 0.0500 | 0.4038 |
| 3 | rs2241766 | G | T | 0.0079 | 0.1333 | 0.2833 | 0.2019 |
| 4 | rs4961 | T | G | 0.0397 | 0.2250 | 0.5333 | 0.2019 |
| 4 | rs1024323 | T | C | 0.6984 | 0.4583 | 0.1583 | 0.4020 |
| 4 | rs1801058 | T | C | 0.0714 | 0.4500 | 0.5167 | 0.3942 |
| 4 | rs1801260 | G | A | 0.1429 | 0.2333 | 0.1083 | 0.2115 |
| 4 | rs1902023 | A | C | 0.4762 | 0.5583 | 0.4417 | 0.2500 |
| 4 | rs4694362 | C | T | 0.8730 | 0.4083 | 0.1833 | 0.1827 |
| 4 | rs4073 | A | T | 0.8492 | 0.4417 | 0.3417 | 0.3173 |
| 4 | rs2231142 | T | G | 0.0159 | 0.0833 | 0.2333 | 0.4804 |
| 4 | rs5743704 | A | C | 0.0000 | 0.0333 | 0.0000 | 0.0000 |
| 5 | rs3846662 | A | G | 0.0476 | 0.4833 | 0.4417 | 0.4519 |
| 5 | rs17238540 | G | T | 0.0873 | 0.0167 | 0.0000 | 0.0000 |
| 5 | rs6196 | G | A | 0.1905 | 0.1500 | 0.0917 | 0.0100 |
| 5 | rs1042713 | A | G | 0.4921 | 0.4083 | 0.4583 | 0.5096 |
| 5 | rs730012 | C | A | 0.0403 | 0.2750 | 0.1333 | 0.1154 |
| 6 | rs1142345 | C | T | 0.0635 | 0.0250 | 0.0250 | 0.0481 |
| 6 | rs1800460 | T | C | 0.0000 | 0.0250 | 0.0000 | 0.0481 |
| 6 | rs12201199 | T | A | 0.4762 | 0.0500 | 0.0250 | 0.0481 |
| 6 | rs1799945 | G | C | 0.0079 | 0.1750 | 0.0417 | 0.0481 |
| 6 | rs1800629 | A | G | 0.0952 | 0.1583 | 0.0167 | 0.0098 |
| 6 | rs361525 | A | G | 0.0714 | 0.1250 | 0.0250 | 0.0385 |
| 6 | rs3093726 | C | T | 0.0556 | 0.1000 | 0.0083 | 0.0098 |
| 6 | rs2227956 | G | A | 0.0000 | 0.2083 | 0.1500 | 0.0289 |
| 6 | rs916344 | C | G | 0.0635 | 0.0917 | 0.0000 | 0.0096 |
| 6 | rs20455 | G | A | 0.8889 | 0.3583 | 0.4333 | 0.1731 |
| 6 | rs833061 | C | T | 0.3333 | 0.4833 | 0.3583 | 0.3846 |
| 6 | rs2010963 | C | G | 0.3333 | 0.3250 | 0.4167 | 0.4038 |
| 6 | rs3025039 | T | C | 0.0556 | 0.1583 | 0.1750 | 0.4327 |
| 6 | rs760370 | G | A | 0.2540 | 0.4000 | 0.2167 | 0.3654 |
| 6 | rs2518224 | C | A | 0.0238 | 0.1250 | 0.0000 | 0.0577 |
| 6 | rs714368 | C | T | 0.4524 | 0.2500 | 0.3917 | 0.1058 |
| 6 | rs2234693 | C | T | 0.4841 | 0.4583 | 0.4000 | 0.2500 |
| 6 | rs1799971 | G | A | 0.0000 | 0.1167 | 0.4167 | 0.1827 |
| 6 | rs4880 | G | A | 0.4206 | 0.4833 | 0.0667 | 0.7692 |
| 6 | rs12208357 | T | C | 0.0000 | 0.0417 | 0.0000 | 0.0000 |
| 6 | rs55918055 | C | T | 0.0000 | 0.0083 | 0.0000 | 0.0000 |
| 6 | rs622342 | C | A | 0.1825 | 0.3750 | 0.1750 | 0.4423 |
| 6 | rs34059508 | A | G | 0.0000 | 0.0167 | 0.0000 | 0.0000 |
| 6 | rs316019 | A | C | 0.2222 | 0.1167 | 0.0833 | 0.0000 |
| 7 | rs37973 | G | A | 0.1984 | 0.4083 | 0.4583 | 0.5962 |
| 7 | rs7793837 | T | A | 0.8889 | 0.2667 | 0.1917 | 0.3942 |
| 7 | rs2227983 | A | G | 0.0952 | 0.3000 | 0.5750 | 0.2115 |
| 7 | rs1045642 | A | G | 0.1190 | 0.4583 | 0.4417 | 0.4808 |
| 7 | rs2229109 | T | C | 0.0000 | 0.0500 | 0.0000 | 0.0000 |
| 7 | rs10276036 | C | T | 0.1905 | 0.3583 | 0.6833 | 0.5481 |
| 7 | rs9282564 | C | T | 0.0000 | 0.0667 | 0.0000 | 0.0000 |
| 7 | rs662 | T | C | 0.2302 | 0.6250 | 0.2750 | 0.4327 |
| 7 | rs10264272 | T | C | 0.2063 | 0.0000 | 0.0000 | 0.0000 |
| 7 | rs776746 | T | C | 0.8175 | 0.1083 | 0.2917 | 0.2981 |
| 7 | rs2242480 | T | C | 0.8492 | 0.1333 | 0.2583 | 0.6923 |
| 7 | rs28371759 | G | A | 0.0000 | 0.0000 | 0.0250 | 0.0000 |
| 7 | rs2246709 | G | A | 0.3810 | 0.2750 | 0.4417 | 0.7308 |
| 7 | rs35599367 | A | G | 0.0000 | 0.0333 | 0.0000 | 0.0000 |
| 7 | rs2740574 | C | T | 0.7222 | 0.0417 | 0.0000 | 0.0673 |
| 7 | rs2278294 | T | C | 0.4048 | 0.3333 | 0.5167 | 0.5288 |
| 7 | rs339097 | G | A | 0.1349 | 0.0000 | 0.0083 | 0.0000 |
| 7 | rs2070744 | C | T | 0.1984 | 0.4917 | 0.1167 | 0.0192 |
| 7 | rs1799983 | T | G | 0.0476 | 0.3417 | 0.1167 | 0.0577 |
| 8 | rs1801279 | A | G | 0.1111 | 0.0000 | 0.0000 | 0.0000 |
| 8 | rs1799929 | T | C | 0.2063 | 0.4333 | 0.0500 | 0.2600 |
| 8 | rs1799930 | A | G | 0.2460 | 0.3000 | 0.2500 | 0.0192 |
| 8 | rs1799931 | A | G | 0.0476 | 0.0167 | 0.1833 | 0.3431 |
| 8 | rs320 | G | T | 0.2937 | 0.2917 | 0.2333 | 0.1442 |
| 8 | rs328 | G | C | 0.0397 | 0.1750 | 0.1250 | 0.0000 |
| 8 | rs1048101 | A | G | 0.1429 | 0.5833 | 0.1000 | 0.6275 |
| 8 | rs4994 | G | A | 0.1349 | 0.1333 | 0.1000 | 0.2596 |
| 8 | rs13266634 | T | C | 0.0635 | 0.2417 | 0.4667 | 0.2788 |
| 8 | rs1799998 | G | A | 0.1667 | 0.4000 | 0.3167 | 0.2596 |
| 9 | rs4986790 | G | A | 0.0714 | 0.0417 | 0.0000 | 0.0000 |
| 9 | rs3842787 | T | C | 0.1508 | 0.0833 | 0.0000 | 0.0000 |
| 10 | rs12248560 | T | C | 0.2143 | 0.2083 | 0.0000 | 0.0000 |
| 10 | rs28399504 | G | A | 0.0000 | 0.0000 | 0.0000 | 0.0000 |
| 10 | rs17884712 | A | G | 0.0318 | 0.0000 | 0.0000 | 0.0000 |
| 10 | rs4986893 | A | G | 0.0000 | 0.0000 | 0.0583 | 0.0000 |
| 10 | rs4244285 | A | G | 0.2222 | 0.1167 | 0.3167 | 0.0577 |
| 10 | rs72558187 | C | T | 0.0000 | 0.0000 | 0.0000 | 0.0000 |
| 10 | rs7900194 | A | G | 0.0556 | 0.0000 | 0.0000 | 0.0000 |
| 10 | rs28371685 | T | C | 0.0318 | 0.0000 | 0.0000 | 0.0000 |
| 10 | rs1057910 | C | A | 0.0000 | 0.0667 | 0.0583 | 0.0192 |
| 10 | rs28371686 | G | C | 0.0159 | 0.0000 | 0.0000 | 0.0000 |
| 10 | rs1058932 | A | G | 0.4206 | 0.1750 | 0.4417 | 0.0577 |
| 10 | rs11572103 | A | T | 0.1825 | 0.0000 | 0.0000 | 0.0000 |
| 10 | rs11572080 | T | C | 0.0079 | 0.1000 | 0.0000 | 0.0000 |
| 10 | rs717620 | T | C | 0.0238 | 0.2000 | 0.2000 | 0.0196 |
| 10 | rs2273697 | A | G | 0.2063 | 0.2333 | 0.1333 | 0.0577 |
| 10 | rs3740066 | T | C | 0.2302 | 0.3500 | 0.2000 | 0.3558 |
| 10 | rs8187710 | A | G | 0.1905 | 0.0833 | 0.0000 | 0.0000 |
| 10 | rs1801252 | G | A | 0.2381 | 0.1333 | 0.1333 | 0.4135 |
| 10 | rs1801253 | G | C | 0.3968 | 0.2500 | 0.2000 | 0.0000 |
| 11 | rs9937 | G | A | 0.1032 | 0.5917 | 0.4833 | 0.2692 |
| 11 | rs5219 | T | C | 0.0000 | 0.2667 | 0.3833 | 0.3269 |
| 11 | rs1800532 | T | G | 0.1190 | 0.4000 | 0.4833 | 0.4423 |
| 11 | rs6265 | T | C | 0.0000 | 0.1667 | 0.4833 | 0.2019 |
| 11 | rs1799963 | A | G | 0.0000 | 0.0167 | 0.0000 | 0.0000 |
| 11 | rs9344 | A | G | 0.1825 | 0.4667 | 0.5167 | 0.2019 |
| 11 | rs1820453 | C | A | 0.2381 | 0.5333 | 0.2667 | 0.3173 |
| 11 | rs6277 | A | G | 0.0476 | 0.5833 | 0.0917 | 0.0000 |
| 11 | rs11214606 | T | C | 0.0000 | 0.0417 | 0.0000 | 0.0000 |
| 11 | rs3135506 | C | G | 0.0318 | 0.0417 | 0.0000 | 0.2404 |
| 11 | rs5128 | G | C | 0.2063 | 0.0917 | 0.2917 | 0.4327 |
| 11 | rs1954787 | T | C | 0.9444 | 0.5333 | 0.1500 | 0.1442 |
| 12 | rs2239128 | T | C | 0.6290 | 0.2417 | 0.3500 | 0.0000 |
| 12 | rs767455 | C | T | 0.3254 | 0.3750 | 0.1667 | 0.1078 |
| 12 | rs4149015 | A | G | 0.0079 | 0.0500 | 0.0833 | 0.0096 |
| 12 | rs2306283 | A | G | 0.1905 | 0.5917 | 0.2583 | 0.3558 |
| 12 | rs11045819 | A | C | 0.0635 | 0.1750 | 0.0000 | 0.0000 |
| 12 | rs4149056 | C | T | 0.0079 | 0.1750 | 0.1083 | 0.2500 |
| 12 | rs2228570 | A | G | 0.1587 | 0.3917 | 0.2750 | 0.5000 |
| 12 | rs2228224 | A | G | 0.1111 | 0.6583 | 0.4083 | 0.1154 |
| 12 | rs2660845 | G | A | 0.3333 | 0.2917 | 0.5667 | 0.6961 |
| 12 | rs56163822 | T | G | 0.0159 | 0.0167 | 0.1833 | 0.0000 |
| 13 | rs10507391 | T | A | 0.0794 | 0.6583 | 0.6333 | 0.4231 |
| 13 | rs6314 | A | G | 0.1032 | 0.0667 | 0.0000 | 0.0096 |
| 13 | rs7997012 | A | G | 0.0000 | 0.3667 | 0.1917 | 0.3558 |
| 13 | rs6311 | T | C | 0.4762 | 0.4417 | 0.4583 | 0.2885 |
| 14 | rs4986938 | T | C | 0.1746 | 0.3667 | 0.1333 | 0.0196 |
| 14 | rs1256049 | T | C | 0.1270 | 0.0500 | 0.3750 | 0.0096 |
| 14 | rs2236225 | A | G | 0.1905 | 0.3750 | 0.2333 | 0.7157 |
| 15 | rs7164902 | A | G | 0.2143 | 0.2833 | 0.4250 | 0.1442 |
| 15 | rs4646 | A | C | 0.3730 | 0.2750 | 0.2833 | 0.9231 |
| 15 | rs1800588 | C | T | 0.4524 | 0.6917 | 0.5583 | 0.1538 |
| 15 | rs1048943 | C | T | 0.0000 | 0.0167 | 0.1750 | 0.5980 |
| 15 | rs762551 | C | A | 0.4524 | 0.3333 | 0.3500 | 0.1827 |
| 15 | rs2272037 | T | C | 0.7778 | 0.4250 | 0.3750 | 0.3269 |
| 16 | rs2230739 | C | T | 0.1667 | 0.3833 | 0.4167 | 0.1538 |
| 16 | rs1799801 | C | T | 0.1587 | 0.3000 | 0.2750 | 0.3558 |
| 16 | rs119774 | T | C | 0.0000 | 0.0667 | 0.0000 | 0.0000 |
| 16 | rs212090 | A | T | 0.1032 | 0.4667 | 0.1667 | 0.6346 |
| 16 | rs5723 | G | C | 0.2143 | 0.2500 | 0.1000 | 0.0096 |
| 16 | rs7294 | T | C | 0.5159 | 0.3500 | 0.1083 | 0.5700 |
| 16 | rs2884737 | C | A | 0.0000 | 0.2833 | 0.0000 | 0.0192 |
| 16 | rs9923231 | T | C | 0.0318 | 0.4167 | 0.8917 | 0.4423 |
| 16 | rs5569 | A | G | 0.0476 | 0.3417 | 0.2083 | 0.4808 |
| 16 | rs5882 | G | A | 0.5714 | 0.2333 | 0.5000 | 0.3846 |
| 16 | rs1800566 | A | G | 0.1190 | 0.2500 | 0.4083 | 0.6346 |
| 16 | rs4673 | A | G | 0.4841 | 0.3583 | 0.0667 | 0.0962 |
| 17 | rs1126667 | A | G | 0.3175 | 0.3500 | 0.4833 | 0.2308 |
| 17 | rs434473 | G | A | 0.1587 | 0.3500 | 0.4750 | 0.0481 |
| 17 | rs799917 | G | A | 0.1190 | 0.5583 | 0.6583 | 0.4902 |
| 17 | rs5918 | C | T | 0.0397 | 0.0833 | 0.0083 | 0.0000 |
| 18 | rs6103 | G | C | 0.4127 | 0.2000 | 0.4750 | 0.5288 |
| 18 | rs763361 | C | T | 0.2778 | 0.4833 | 0.5500 | 0.3431 |
| 19 | rs1799969 | A | G | 0.0000 | 0.0833 | 0.0000 | 0.2404 |
| 19 | rs688 | T | C | 0.0159 | 0.4583 | 0.1667 | 0.3173 |
| 19 | rs14158 | A | G | 0.1190 | 0.1917 | 0.3917 | 0.4327 |
| 19 | rs2108622 | T | C | 0.0794 | 0.3667 | 0.2167 | 0.0962 |
| 19 | rs4805924 | T | C | 0.0079 | 0.0167 | 0.0833 | 0.1827 |
| 19 | rs9403 | C | G | 0.3492 | 0.6583 | 0.2667 | 0.2596 |
| 19 | rs8099917 | G | T | 0.0714 | 0.1833 | 0.0667 | 0.4314 |
| 19 | rs4986891 | A | C | 0.0000 | 0.0000 | 0.0000 | 0.0000 |
| 19 | rs8192709 | T | C | 0.0476 | 0.0500 | 0.0417 | 0.0000 |
| 19 | rs3745274 | T | G | 0.2857 | 0.2167 | 0.2083 | 0.4423 |
| 19 | rs28399499 | C | T | 0.0794 | 0.0000 | 0.0000 | 0.0000 |
| 19 | rs25487 | T | C | 0.0873 | 0.4083 | 0.2083 | 0.2692 |
| 19 | rs13181 | G | T | 0.1984 | 0.3333 | 0.0583 | 0.0769 |
| 19 | rs238406 | T | G | 0.0556 | 0.5167 | 0.5000 | 0.5769 |
| 19 | rs3212948 | G | C | 0.0318 | 0.7167 | 0.3333 | 0.1538 |
| 20 | rs1127354 | A | C | 0.0318 | 0.1083 | 0.1917 | 0.0000 |
| 20 | rs7270101 | C | A | 0.0556 | 0.1583 | 0.0000 | 0.0000 |
| 21 | rs1056892 | A | G | 0.5794 | 0.4000 | 0.3167 | 0.1635 |
| 21 | rs1051266 | T | C | 0.7302 | 0.4417 | 0.5000 | 0.2885 |
| 22 | rs6269 | G | A | 0.2937 | 0.5000 | 0.3250 | 0.0769 |
| 22 | rs4680 | A | G | 0.3095 | 0.3917 | 0.2583 | 0.2885 |
| 22 | rs4646316 | T | C | 0.1746 | 0.3333 | 0.3333 | 0.0686 |
| 22 | rs9332377 | T | C | 0.3333 | 0.1667 | 0.0000 | 0.0096 |
| 22 | rs165599 | A | G | 0.2857 | 0.6750 | 0.6083 | 0.2500 |
| 22 | rs5760410 | G | A | 0.3889 | 0.4417 | 0.3750 | 0.3365 |
|  |  |  |  |  |  |  |  |
| **VIP panel** | | | | | | | |
| CHR | SNP | A1 | A2 | AFR | EUR | EAS | AMR |
| 1 | rs1801131 | G | T | 0.1190 | 0.3083 | 0.2000 | 0.0673 |
| 1 | rs890293 | A | C | 0.2063 | 0.0583 | 0.0333 | 0.0000 |
| 1 | rs1801160 | T | C | 0.0079 | 0.0833 | 0.0333 | 0.0096 |
| 1 | rs1801159 | C | T | 0.1746 | 0.1833 | 0.1500 | 0.3846 |
| 1 | rs1801158 | T | C | 0.0000 | 0.0083 | 0.0000 | 0.0000 |
| 1 | rs1801265 | G | A | 0.4841 | 0.2333 | 0.0583 | 0.1731 |
| 1 | rs6025 | T | C | 0.0000 | 0.0167 | 0.0000 | 0.0000 |
| 1 | rs5275 | G | A | 0.7222 | 0.2750 | 0.1833 | 0.3173 |
| 1 | rs20417 | G | C | 0.4921 | 0.1000 | 0.0417 | 0.2019 |
| 1 | rs689466 | C | T | 0.0714 | 0.2083 | 0.4750 | 0.4615 |
| 2 | rs4148323 | A | G | 0.0000 | 0.0000 | 0.1250 | 0.0385 |
| 3 | rs7626962 | T | G | 0.0714 | 0.0000 | 0.0000 | 0.0000 |
| 3 | rs1805124 | C | T | 0.2937 | 0.2500 | 0.1083 | 0.2500 |
| 3 | rs6791924 | A | G | 0.1349 | 0.0083 | 0.0000 | 0.0000 |
| 3 | rs3814055 | T | C | 0.2857 | 0.3667 | 0.2167 | 0.2788 |
| 3 | rs2046934 | G | A | 0.1429 | 0.1917 | 0.1250 | 0.0673 |
| 3 | rs1065776 | T | C | 0.2540 | 0.0250 | 0.0500 | 0.1442 |
| 3 | rs701265 | G | A | 0.8095 | 0.1333 | 0.2417 | 0.3269 |
| 4 | rs975833 | C | G | 0.2302 | 0.3500 | 0.8333 | 0.0096 |
| 4 | rs2066702 | A | G | 0.2143 | 0.0000 | 0.0000 | 0.0000 |
| 4 | rs1229984 | T | C | 0.0000 | 0.0417 | 0.7417 | 0.0000 |
| 4 | rs698 | C | T | 0.0873 | 0.3417 | 0.0583 | 0.3654 |
| 5 | rs3846662 | A | G | 0.0476 | 0.4833 | 0.4417 | 0.4519 |
| 5 | rs17238540 | G | T | 0.0873 | 0.0167 | 0.0000 | 0.0000 |
| 5 | rs1042713 | A | G | 0.4921 | 0.4083 | 0.4583 | 0.5096 |
| 5 | rs1042714 | G | C | 0.1210 | 0.4333 | 0.0833 | 0.0000 |
| 5 | rs1800888 | T | C | 0.0000 | 0.0167 | 0.0000 | 0.0000 |
| 6 | rs1142345 | C | T | 0.0635 | 0.0250 | 0.0250 | 0.0481 |
| 6 | rs1800460 | T | C | 0.0000 | 0.0250 | 0.0000 | 0.0481 |
| 6 | rs12208357 | T | C | 0.0000 | 0.0417 | 0.0000 | 0.0000 |
| 6 | rs34130495 | A | G | 0.0000 | 0.0250 | 0.0000 | 0.0000 |
| 6 | rs34059508 | A | G | 0.0000 | 0.0167 | 0.0000 | 0.0000 |
| 7 | rs2066853 | A | G | 0.4841 | 0.1250 | 0.4167 | 0.1346 |
| 7 | rs1045642 | A | G | 0.1190 | 0.4583 | 0.4417 | 0.4808 |
| 7 | rs1128503 | A | G | 0.1111 | 0.3583 | 0.6833 | 0.5481 |
| 7 | rs10264272 | T | C | 0.2063 | 0.0000 | 0.0000 | 0.0000 |
| 7 | rs776746 | T | C | 0.8175 | 0.1083 | 0.2917 | 0.2981 |
| 7 | rs4986910 | G | A | 0.0000 | 0.0083 | 0.0000 | 0.0096 |
| 7 | rs4986908 | G | C | 0.0000 | 0.0083 | 0.0000 | 0.0000 |
| 7 | rs4986907 | T | C | 0.0159 | 0.0083 | 0.0000 | 0.0000 |
| 7 | rs2740574 | C | T | 0.7222 | 0.0417 | 0.0000 | 0.0673 |
| 7 | rs36210421 | A | C | 0.0000 | 0.0167 | 0.0000 | 0.0000 |
| 7 | rs1805123 | G | T | 0.0000 | 0.3000 | 0.0500 | 0.0000 |
| 7 | rs3807375 | C | T | 0.2222 | 0.7000 | 0.2083 | 0.0865 |
| 8 | rs4271002 | C | G | 0.0794 | 0.1583 | 0.2417 | 0.3462 |
| 8 | rs1801279 | A | G | 0.1111 | 0.0000 | 0.0000 | 0.0000 |
| 8 | rs1799929 | T | C | 0.2063 | 0.4333 | 0.0500 | 0.2600 |
| 8 | rs1799930 | A | G | 0.2460 | 0.3000 | 0.2500 | 0.0192 |
| 8 | rs1208 | G | A | 0.3413 | 0.4250 | 0.0500 | 0.2885 |
| 8 | rs1799931 | A | G | 0.0476 | 0.0167 | 0.1833 | 0.3431 |
| 8 | rs1495741 | G | A | 0.4603 | 0.2417 | 0.4917 | 0.3750 |
| 10 | rs2115819 | A | G | 0.8016 | 0.5750 | 0.1833 | 0.3846 |
| 10 | rs12248560 | T | C | 0.2143 | 0.2083 | 0.0000 | 0.0000 |
| 10 | rs4986893 | A | G | 0.0000 | 0.0000 | 0.0583 | 0.0000 |
| 10 | rs4244285 | A | G | 0.2222 | 0.1167 | 0.3167 | 0.0577 |
| 10 | rs1057910 | C | A | 0.0000 | 0.0667 | 0.0583 | 0.0192 |
| 10 | rs11572103 | A | T | 0.1825 | 0.0000 | 0.0000 | 0.0000 |
| 10 | rs11572080 | T | C | 0.0079 | 0.1000 | 0.0000 | 0.0000 |
| 10 | rs7909236 | T | G | 0.0000 | 0.2917 | 0.0417 | 0.3077 |
| 10 | rs17110453 | C | A | 0.0079 | 0.1083 | 0.3833 | 0.0577 |
| 10 | rs1801252 | G | A | 0.2381 | 0.1333 | 0.1333 | 0.4135 |
| 10 | rs1801253 | G | C | 0.3968 | 0.2500 | 0.2000 | 0.0000 |
| 10 | rs3813867 | C | G | 0.0873 | 0.0333 | 0.1583 | 0.4327 |
| 10 | rs2031920 | T | C | 0.0000 | 0.0333 | 0.1583 | 0.4314 |
| 10 | rs6413432 | A | T | 0.0635 | 0.1167 | 0.2167 | 0.4216 |
| 10 | rs2070676 | G | C | 0.7143 | 0.1250 | 0.1750 | 0.0385 |
| 11 | rs5219 | T | C | 0.0000 | 0.2667 | 0.3833 | 0.3269 |
| 11 | rs1695 | G | A | 0.4762 | 0.3083 | 0.0750 | 0.4804 |
| 11 | rs1138272 | T | C | 0.0000 | 0.0667 | 0.0000 | 0.0000 |
| 11 | rs1800497 | A | G | 0.3651 | 0.2333 | 0.3833 | 0.6250 |
| 11 | rs6277 | A | G | 0.0476 | 0.5833 | 0.0917 | 0.0000 |
| 11 | rs1801028 | C | G | 0.0000 | 0.0167 | 0.0250 | 0.0673 |
| 12 | rs4149015 | A | G | 0.0079 | 0.0500 | 0.0833 | 0.0096 |
| 12 | rs2306283 | A | G | 0.1905 | 0.5917 | 0.2583 | 0.3558 |
| 12 | rs4149056 | C | T | 0.0079 | 0.1750 | 0.1083 | 0.2500 |
| 12 | rs7975232 | A | C | 0.6825 | 0.6000 | 0.3083 | 0.3333 |
| 12 | rs1544410 | T | C | 0.2698 | 0.4500 | 0.0667 | 0.1275 |
| 12 | rs2239185 | A | G | 0.6508 | 0.6000 | 0.3000 | 0.3365 |
| 12 | rs1540339 | T | C | 0.2540 | 0.3250 | 0.6833 | 0.3431 |
| 12 | rs2239179 | C | T | 0.2778 | 0.4500 | 0.2167 | 0.2692 |
| 12 | rs3782905 | C | G | 0.2222 | 0.3500 | 0.1250 | 0.2059 |
| 12 | rs2228570 | A | G | 0.1587 | 0.3917 | 0.2750 | 0.5000 |
| 12 | rs4516035 | C | T | 0.0079 | 0.4167 | 0.0083 | 0.3077 |
| 12 | rs11568820 | T | C | 0.9444 | 0.2250 | 0.4583 | 0.0192 |
| 15 | rs2069514 | A | G | 0.2937 | 0.0083 | 0.2250 | 0.6731 |
| 15 | rs12720461 | T | C | 0.0000 | 0.0000 | 0.0000 | 0.0000 |
| 15 | rs762551 | C | A | 0.4524 | 0.3333 | 0.3500 | 0.1827 |
| 16 | rs750155 | T | C | 0.3276 | 0.4000 | 0.4167 | 0.8824 |
| 16 | rs3760091 | G | C | 0.3909 | 0.5083 | 0.3167 | 0.1154 |
| 16 | rs7294 | T | C | 0.5159 | 0.3500 | 0.1083 | 0.5700 |
| 16 | rs9923231 | T | C | 0.0318 | 0.4167 | 0.8917 | 0.4423 |
| 16 | rs1800566 | A | G | 0.1190 | 0.2500 | 0.4083 | 0.6346 |
| 19 | rs2108622 | T | C | 0.0794 | 0.3667 | 0.2167 | 0.0962 |
| 19 | rs28399454 | T | C | 0.1587 | 0.0000 | 0.0000 | 0.0000 |
| 19 | rs1801272 | T | A | 0.0000 | 0.0417 | 0.0000 | 0.0000 |
| 19 | rs28399433 | C | A | 0.0635 | 0.0417 | 0.3417 | 0.2596 |
| 19 | rs3745274 | T | G | 0.2857 | 0.2167 | 0.2083 | 0.4423 |
| 19 | rs28399499 | C | T | 0.0794 | 0.0000 | 0.0000 | 0.0000 |
| 20 | rs5629 | T | G | 0.1111 | 0.2833 | 0.3250 | 0.2212 |
| 21 | rs12659 | A | G | 0.4444 | 0.4583 | 0.5083 | 0.2843 |
| 21 | rs1131596 | A | G | 0.2143 | 0.5583 | 0.5000 | 0.7115 |
| 22 | rs4680 | A | G | 0.3095 | 0.3917 | 0.2583 | 0.2885 |
|  |  |  |  |  |  |  |  |
| **DMET panel** | | | | | | | |
| CHR | SNP | A1 | A2 | AFR | EUR | EAS | AMR |
| 1 | rs11211402 | C | T | 0.5714 | 0.1083 | 0.2250 | 0.2019 |
| 1 | rs1800822 | T | C | 0.0556 | 0.1167 | 0.2667 | 0.4808 |
| 1 | rs1126692 | G | A | 0.5159 | 0.1250 | 0.0167 | 0.0000 |
| 1 | rs1051740 | C | T | 0.1746 | 0.2833 | 0.4833 | 0.4712 |
| 2 | rs2295475 | A | G | 0.0238 | 0.3083 | 0.3917 | 0.4615 |
| 2 | rs3828193 | T | G | 0.0159 | 0.5333 | 0.6667 | 0.5865 |
| 2 | rs1402467 | G | C | 0.9127 | 0.2000 | 0.1833 | 0.0096 |
| 2 | rs4668115 | T | C | 0.1190 | 0.2833 | 0.2250 | 0.6154 |
| 2 | rs1048013 | T | C | 0.0873 | 0.5417 | 0.2917 | 0.2404 |
| 2 | rs4124874 | T | G | 0.1111 | 0.5333 | 0.6500 | 0.4804 |
| 3 | rs9833097 | A | G | 0.0714 | 0.1000 | 0.0000 | 0.3462 |
| 3 | rs4679028 | A | G | 0.5873 | 0.2250 | 0.2750 | 0.0962 |
| 3 | rs6771233 | G | T | 0.7460 | 0.5583 | 0.3500 | 0.1154 |
| 3 | rs6785049 | A | G | 0.0079 | 0.6917 | 0.4167 | 0.6923 |
| 3 | rs2293616 | A | G | 0.5317 | 0.3833 | 0.7583 | 0.0385 |
| 3 | rs3755739 | A | G | 0.7540 | 0.1750 | 0.3833 | 0.5769 |
| 3 | rs562 | T | C | 0.2302 | 0.5000 | 0.5500 | 0.5686 |
| 4 | rs1902023 | A | C | 0.4762 | 0.5583 | 0.4417 | 0.2500 |
| 4 | rs7662029 | A | G | 0.1190 | 0.5250 | 0.2917 | 0.1250 |
| 4 | rs11249454 | C | T | 0.0238 | 0.1583 | 0.2500 | 0.5577 |
| 4 | rs4643786 | C | T | 0.7143 | 0.0667 | 0.0500 | 0.0769 |
| 4 | rs3756067 | A | G | 0.2778 | 0.3417 | 0.3667 | 0.1635 |
| 4 | rs1154400 | C | T | 0.3571 | 0.3000 | 0.0000 | 0.0000 |
| 4 | rs12512110 | T | G | 0.0397 | 0.0750 | 0.0833 | 0.5481 |
| 4 | rs1442477 | T | C | 0.3016 | 0.0083 | 0.1250 | 0.1058 |
| 5 | rs1050152 | T | C | 0.0000 | 0.3500 | 0.0000 | 0.0000 |
| 6 | rs6901410 | C | T | 0.7460 | 0.1417 | 0.0333 | 0.0096 |
| 6 | rs2242416 | G | A | 0.0635 | 0.5917 | 0.6083 | 0.2212 |
| 6 | rs9369629 | C | G | 0.0397 | 0.0500 | 0.0833 | 0.5385 |
| 6 | rs512795 | C | T | 0.6667 | 0.1083 | 0.3500 | 0.6346 |
| 6 | rs628031 | A | G | 0.2381 | 0.3667 | 0.2250 | 0.0289 |
| 6 | rs624249 | A | C | 0.3333 | 0.4083 | 0.1167 | 0.0096 |
| 7 | rs2066853 | A | G | 0.4841 | 0.1250 | 0.4167 | 0.1346 |
| 7 | rs1202283 | A | G | 0.0079 | 0.4583 | 0.4083 | 0.4231 |
| 7 | rs2242480 | T | C | 0.8492 | 0.1333 | 0.2583 | 0.6923 |
| 7 | rs1049793 | G | C | 0.5476 | 0.2083 | 0.5417 | 0.7212 |
| 8 | rs13251066 | G | A | 0.1349 | 0.3667 | 0.3250 | 0.8365 |
| 8 | rs3750266 | A | G | 0.7302 | 0.3583 | 0.3500 | 0.2212 |
| 9 | rs13959 | A | G | 0.2302 | 0.4167 | 0.4333 | 0.5673 |
| 9 | rs2636889 | C | T | 0.2937 | 0.0000 | 0.0000 | 0.0000 |
| 10 | rs4148945 | T | C | 0.0161 | 0.5250 | 0.0917 | 0.2059 |
| 10 | rs9285726 | A | T | 0.0318 | 0.3167 | 0.0500 | 0.0769 |
| 10 | rs10882140 | G | T | 0.3333 | 0.4333 | 0.3000 | 0.3077 |
| 10 | rs2070673 | A | T | 0.8095 | 0.1667 | 0.3750 | 0.4804 |
| 11 | rs757110 | C | A | 0.0000 | 0.2583 | 0.4083 | 0.3269 |
| 11 | rs2078267 | T | C | 0.0635 | 0.6000 | 0.0000 | 0.0096 |
| 11 | rs2852425 | C | T | 0.6825 | 0.2167 | 0.1833 | 0.2115 |
| 12 | rs3764006 | G | A | 0.8016 | 0.0833 | 0.2833 | 0.3750 |
| 12 | rs2291075 | T | C | 0.5952 | 0.4250 | 0.4083 | 0.2596 |
| 12 | rs903247 | C | T | 0.4127 | 0.3000 | 0.6333 | 0.6058 |
| 12 | rs886205 | A | G | 0.1825 | 0.7750 | 0.1500 | 0.5577 |
| 13 | rs2277448 | G | T | 0.7302 | 0.3250 | 0.4500 | 0.3846 |
| 14 | rs8018462 | A | G | 0.5714 | 0.5083 | 0.2167 | 0.2596 |
| 14 | rs910795 | G | A | 0.5873 | 0.1333 | 0.3167 | 0.0000 |
| 15 | rs1060896 | A | C | 0.0873 | 0.5833 | 0.0500 | 0.0192 |
| 15 | rs700518 | C | T | 0.1587 | 0.4500 | 0.4167 | 0.0192 |
| 15 | rs2242046 | A | G | 0.0159 | 0.4583 | 0.0583 | 0.0192 |
| 16 | rs246221 | C | T | 0.7222 | 0.3083 | 0.4250 | 0.1827 |
| 16 | rs11150606 | C | T | 0.0000 | 0.0167 | 0.8083 | 0.4231 |
| 16 | rs4783745 | G | A | 0.5873 | 0.1083 | 0.1750 | 0.4231 |
| 16 | rs2641806 | A | G | 0.1984 | 0.7833 | 0.0500 | 0.0962 |
| 16 | rs1060253 | C | G | 0.0794 | 0.2583 | 0.6000 | 0.7692 |
| 19 | rs2279344 | G | A | 0.1984 | 0.3917 | 0.2583 | 0.0096 |
| 19 | rs2302948 | T | C | 0.2143 | 0.1667 | 0.0833 | 0.1538 |
| 20 | rs2296241 | A | G | 0.4841 | 0.5250 | 0.4417 | 0.3654 |
| 21 | rs1541290 | A | G | 0.1190 | 0.4917 | 0.6250 | 0.5673 |
| 22 | rs138057 | G | A | 0.3333 | 0.1750 | 0.1083 | 0.0000 |

| S**upplementary Table 2. Fst estimates for the PGx panels** | | | | | |
| --- | --- | --- | --- | --- | --- |
| **SNP panels** | **Mean Fst estimate** | | | | |
| **Preemptive-PG-x** |  | AFR | EUR | EAS | AMR |
|  | AFR | 0.0000 | 0.1180 | 0.1414 | 0.1568 |
|  | EUR | 0.1180 | 0.0000 | 0.0769 | 0.1044 |
|  | EAS | 0.1414 | 0.0769 | 0.0000 | 0.0917 |
|  | AMR | 0.1568 | 0.1044 | 0.0917 | 0.0000 |
|  |  |  |  |  |  |
| **DMET** |  | AFR | EUR | EAS | AMR |
|  | AFR | 0.0000 | 0.2558 | 0.2131 | 0.2762 |
|  | EUR | 0.2558 | 0.0000 | 0.1189 | 0.1931 |
|  | EAS | 0.2131 | 0.1189 | 0.0000 | 0.1059 |
|  | AMR | 0.2762 | 0.1931 | 0.1059 | 0.0000 |
|  |  |  |  |  |  |
| **VIP** |  | AFR | EUR | EAS | AMR |
|  | AFR | 0.0000 | 0.1219 | 0.1571 | 0.1652 |
|  | EUR | 0.1219 | 0.0000 | 0.0970 | 0.0980 |
|  | EAS | 0.1571 | 0.0970 | 0.0000 | 0.1104 |
|  | AMR | 0.1652 | 0.0980 | 0.1104 | 0.0000 |
|  |  |  |  |  |  |
